# Ion Chemistry in Dielectric Barrier Discharge Ionization: Recent Advances in Direct Gas Phase Analyses

**DOI:** 10.1002/mas.21914

**Published:** 2024-11-06

**Authors:** Kseniya Dryahina, Miroslav Polášek, Juraj Jašík, Kristýna Sovová, Patrik Španěl

**Affiliations:** ^1^ J. Heyrovský Institute of Physical Chemistry Czech Academy of Sciences Prague Czechia

**Keywords:** dielectric barrier discharge ionization, direct analyses, gas phase, ion chemistry

## Abstract

Dielectric barrier discharge ionization (DBDI) sources, employing low‐temperature plasma, have emerged as sensitive and efficient ionization tools with various atmospheric pressure ionization processes. In this review, we summarize a historical overview of the development of DBDI, highlighting key principles of gas‐phase ion chemistry and the mechanisms underlying the ionization processes within the DBDI source. These processes start with the formation of reagent ions or metastable atoms from the discharge gas, which depends on the nature of the gas (helium, nitrogen, air) and on the presence of water vapor or other compounds or dopants. The processes of ionizing the analyte molecules are summarized, including Penning ionization, electron transfer, proton transfer and ligand switching from secondary hydrated hydronium ions. Presently, the DBDI‐MS methods face a challenge in the accurate quantification of gaseous analytes, limiting its broader application in biological, environmental, and medical realms where relative quantification using standards is inherently complex for gaseous matrices. Finally, we propose future avenues of research to enhance the analytical capabilities of DBDI‐MS.

**Abbreviations**
ACaPI: active capillary plasma ionization
AMS: ambient mass spectrometry
APCI: atmospheric pressure chemical ionization
CA‐FμTP: controlled atmosphere flexible microtube plasma
C‐DBD: capillary DBD, also known as a cylindrical DBD
DART: direct analysis in real‐time
DBD: dielectric barrier discharge
DBDI: dielectric‐barrier‐discharge ion source
FAPA: flowing atmospheric‐pressure afterglow
FμTP: flexible microtube plasma
LP‐DBDI: low‐pressure dielectric‐barrier‐discharge ion source
LTP: low‐temperature plasma
ND‐DBDI: neutral desorption DBDI
TPI: tube plasma ionization

## 1 Introduction

Renato Zenobi, to whom we dedicate this review, is a true Renaissance personality when it comes to the variety and diversity of his substantial contributions to analytical chemistry. His work spans optical spectroscopy and numerous innovative advancements in mass spectrometry. Among these, dielectric barrier discharge ionization (DBDI) is gaining prominence for its capability to ionize vaporized and gaseous samples softly (Gyr et al. 2018). DBDI extends the range of available atmospheric pressure ionization techniques. These include well‐established techniques such as atmospheric pressure chemical ionization (APCI), atmospheric pressure photoionization (APPI), and the more recently introduced secondary electrospray ionization (SESI) (Ayala‐Cabrera et al. 2023). The recent widespread use of this approach has been well‐documented in several comprehensive reviews outlining the current development state (Gross 2014; Guo et al. 2015b; Brandt et al. 2017; Li et al. 2018; Ayala‐Cabrera et al. 2023; Yue et al. 2023; Pape and Schmitz 2024). Our present review will concentrate on the status of the development and commercialization of DBDI, focusing on understanding the underlying ion chemistry that leads to the formation of observable product ions in its various applications for gas phase samples, whether they are natively gaseous, desorbed from surfaces, or collected as headspace.

Several reviews have already covered the use of nitrogen (N₂) and helium (He) as discharge gases in DBDI (Guo et al. 2015a; Pape and Schmitz 2024). The present review will build on that foundation by investigating the specifics of ionization mechanisms occurring in the presence of air constituents, including water vapor. This will provide a deeper understanding of how various positive and negative ion species are formed from different analytes.

Historically, Siemens introduced the concept of dielectric barrier discharge in 1857 for ozone production (Siemens 1857; Almasian et al. 2010). However, it wasn't until the early 2000s that DBD began to be used as a low‐temperature plasma ionization technique in analytical mass spectrometry. The first application of DBD in analytical chemistry was reported by Miclea et al. in 2001, who used DBD as a plasma source for analytical atomic spectroscopy (Miclea et al. 2001). This configuration had two flat glass plates with aluminum electrodes forming a 1 mm wide and 6 cm long discharge channel. Ar or He were used as discharge gases to excite molecules like halomethanes. A few years later, the same DBD arrangement was tested as an element‐selective diode laser atomic absorption detector for gas chromatography of halocarbons (Kunze et al. 2003; Michels et al. 2007).

DBDI was used as an ionization source for ion mobility spectrometry by Franzke and colleagues in 2006. (Franzke and Miclea 2006; Michels et al. 2007). DBDI for surface desorption and ionization for mass spectrometry was introduced by Xinrong Zhang et al. at Tsinghua University in 2007 (Na et al. 2007). The same group, in collaboration with Cooks, used DBD as a source of low‐temperature plasma (LTP) in a study aiming to elucidate Birch reduction (Na et al. 2009). This was later patented and commercialized (Zhao and Wen 2015).

Today, DBDI technology has been commercialized and is available in products like the DBDI‐100 and SICRIT ion sources. These devices are designed as add‐ons to conventional mass analyzers, offering high sensitivity and minimal sample preparation. This makes them highly suitable for real‐time, in situ analysis across various fields, including environmental monitoring, food safety, and clinical diagnostics.

Despite the progress made, there is still much to learn about the ionic reactions involved in DBDI. Understanding this chemistry is crucial for optimizing the methods, assuring quantitative accuracy, and expanding the range of applications. This review aims to provide a comprehensive overview of the ion chemistry involved in DBDI, highlighting recent advancements and identifying areas where further research is needed.

## 2 DBDI Categorization

Recent reviews (Gross 2014; Guo et al. 2015b; Brandt et al. 2017; Li et al. 2018; Ayala‐Cabrera et al. 2023; Yue et al. 2023; Pape and Schmitz 2024) cover details of specific DBDI instrumentation, and we do not intend to replicate this information. In this article, our focus will be on delivering a summary overview of the fundamental categories of sources relevant to ion‐molecule chemistry in the gas phase, which is the primary subject of this review. In the first section, we will categorize the basic types of DBD discharges, emphasizing their construction configurations and physical boundary conditions. In the second section, we will discuss the main categories of ion sources utilizing DBD discharge, focusing on the ionization of samples in the gaseous state (gases, vapors, headspace, and even GC outputs).

### 2.1 DBD Plasma Generation

DBD consists of a ground electrode, a high‐voltage electrode, and dielectric material in between. Electrodes are connected to a high voltage (HV) alternating current (AC) generator. The geometric configuration of the electrodes and dielectric can be very different. The most practical for MS ion sources is a cylindrical symmetry, where the dielectric barrier is usually in the form of a tube. The cylindrical DBD setup forms an enclosed discharge space, which makes it advantageous in the applications of gas conversion and ionization (Niu et al. 2021).

DBD can produce a low‐temperature plasma at atmospheric pressure, yielding a substantial amount of chemically active species. The literature describes two basic types of discharge mechanisms: filamentary and homogeneous. The filamentary mechanism, based on streamer theory, involves the creation of regions of high electric field around the head of the electron avalanche due to space charge, allowing free electrons to multiply efficiently. This process results in the formation of tiny breakdown channels, known as micro‐discharges or filaments, within the dielectric material. On the other hand, the homogeneous mechanism, explained by the Townsend mechanism, involves the secondary emission of electrons from the cathode, the dielectric surface, or a metallic electrode, depending on the specific configuration. The type of the mechanism varies depending on factors such as voltage, gas gap distance, pressure, and temperature (Hodges et al. 1985). High‐energy electrons collide with discharge gas particles present in the gap, creating plasma consisting of free electrons, positive and negative ions, molecules, and atoms. It is low‐temperature, nonequilibrium plasma, where electrons have much higher energy than other components of plasma. However, fast electrons are only present for very brief periods (~µs) during the electric breakdown, which, in the homogeneous mode, occurs once per AC half‐period. In the filament mode, these breakdowns occur more frequently and randomly. Fast electrons are absent during the relatively long intervals between two discharge pulses within the AC cycle. However, thermalized electrons and chemically active species, which play a crucial role in soft chemical ionization, remain present (Müller et al. 2013).

**Figure 1:**
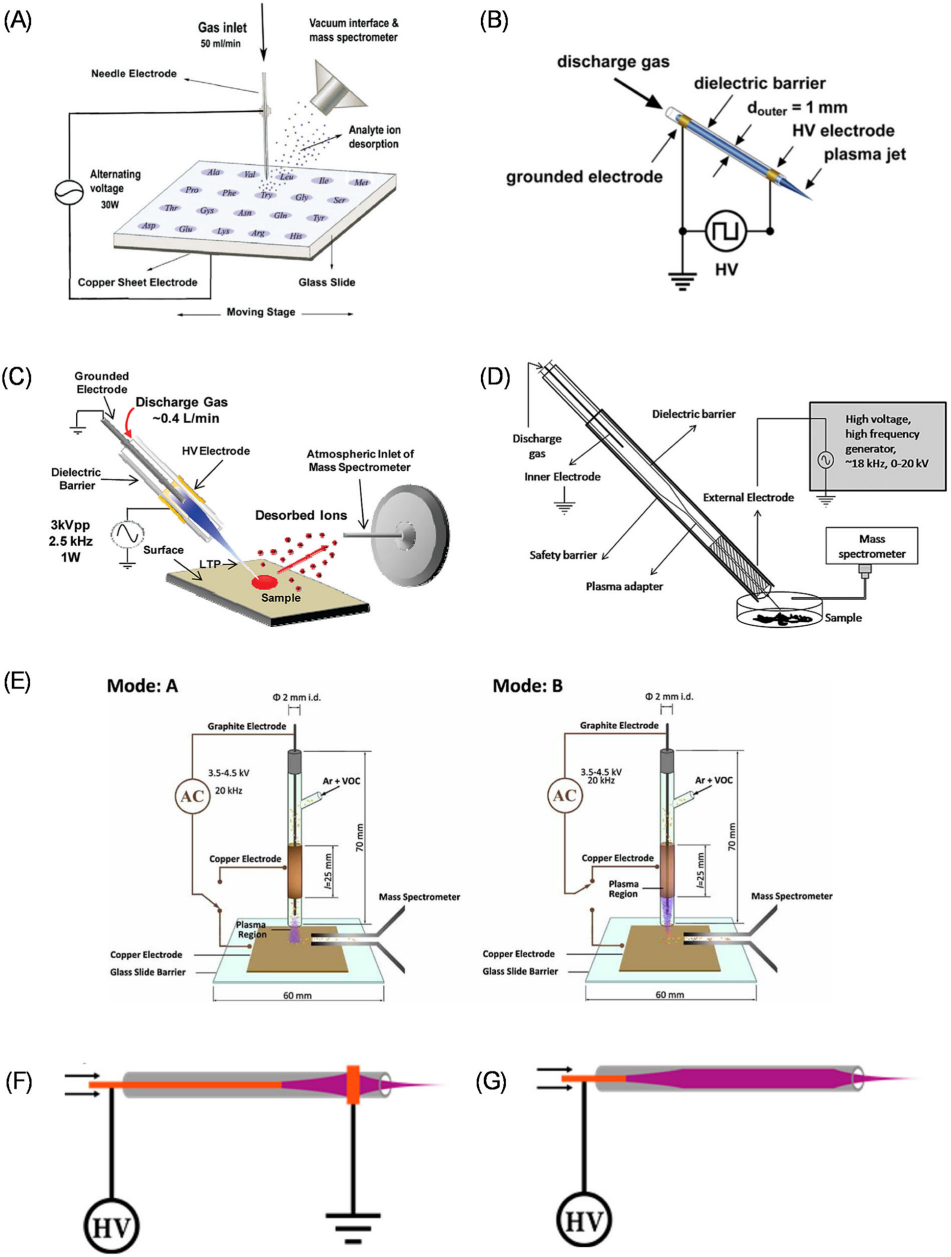
Different DBDI geometries: (A) Pin‐plate DBDI. Adapted with permission from Na et al. (2007). Copyright 2007 American Chemical Society. (B) C‐ DBD ion source. Adapted with permission from Klute et al. (2017). Copyright 2017 American Chemical Society. (C) LTP ion source. Adapted with permission from Harper et al. (2008). Copyright 2008 American Chemical Society. (D) Adjustable LTP probe. Adapted with permission from Martínez‐Jarquín and Winkler (2013). Copyright 2013 John Wiley & Sons, Ltd. (E) Dual‐mode LTP source. Adapted with permission from Almasian et al. (2010). Copyright 2010 John Wiley & Sons Ltd. (F) iLTP. Adapted with permission from Brandt et al. (2018). Copyright 2018 American Chemical Society. (G) FµTP Adapted with permission from Brandt et al. (2018). Copyright 2018 American Chemical Society. [Color figure can be viewed at wileyonlinelibrary.com]

Due to the dielectric barrier, DBD can only be operated with AC voltage. Typical amplitudes are a few kV and frequencies in the kHz to MHz range. The type of AC voltage waveform does not significantly influence ion abundances, which allows the use of a square waveform. This waveform is less demanding on the generator's construction and consumes much less power than sinusoidal or triangular generators (Dumlao et al. 2017b). Different DBDI geometries are illustrated in Figure 1 and summarized next.

The Pin‐plate DBDI (Figure 1A), where the discharge gas flows through a needle electrode with a grounded copper sheet as a counter electrode and glass with the sample acting as a dielectric barrier, marks the first use of DBD as an ion source for MS (Na et al. 2007).

The capillary DBD (Figure 1B), also known as a cylindrical DBD (C‐DBD) or DBDI source, features two annular electrodes wrapped around the dielectric tube (Michels et al. 2007). This configuration is called a full dielectric barrier discharge because the dielectric barrier separates both electrodes from the plasma (Klute et al. 2017).

The LTP ion source (Figure 1C), or LTP probe, consists of a glass tube serving as the dielectric, with discharge gas flowing through a grounded stainless steel needle electrode. An HV AC generator powers an external annular electrode. The steel needle electrode interacts with the plasma. Therefore, it is called a half‐dielectric barrier discharge design. The plasma jet extending beyond the glass tube interacts directly with the sample (Harper et al. 2008).

The adjustable LTP probe (Figure 1D) design has an adjustable output temperature and variable beam diameter. The grounded internal electrode and external annular electrode are driven by an HV AC generator. The plasma is guided through an internal second tube, and changing the insert can modify the plasma beam diameter. The input voltage controls the LTP temperature (Martínez‐Jarquín and Winkler 2013).

The dual‐mode LTP source (Figure 1E) is similar to the LTP probe, but pencil graphite is used as an inner electrode. Two configurations are possible: bar‐plate (copper plate as a counter electrode) and coaxial bar‐cylinder (external annular electrode as a counter electrode). The graphite electrode can act as a sample adsorbent – adsorption of the selected VOCs onto the surface of the graphite electrode in a headspace and direct desorption and ionization of the samples by LTPMS (Almasian et al. 2010).

Especially interesting variants of DBDI are the Inverse LTP (iLTP) (Figure 1F) and Flexible‐microtube plasma (FµTP) (Figure 1G) ion sources. A systematic comparative study of DBDI and LTP configurations led to the discovery of significant differences in ionization efficiency when the pin electrode was grounded or disconnected (floating). The optical emission analysis showed that the LTP configuration generates a plasma discharge with a greater amount of charged species and more emission compared to a similar DBDI configuration. However, MS measurements indicated that the increased emission in the LTP configuration does not directly result in better ionization efficiency, but actually, the ion yield of the LTP configuration is more than one order of magnitude less than that of a comparable DBDI configuration, making it analytically less efficient (Klute et al. 2017). The reduced ion transport efficiency from the discharge to the high vacuum of the mass analyzer causes a lower ion yield in the LTP configuration. This happens because the difference in electric potential between the plasma potential and the transfer capillary, which transports the ions from the atmospheric pressure region, is smaller in LTP than in DBDI. In the DBDI setup, the dielectric barrier prevents charged particles from reaching the conducting ring electrodes, acting as a shield. However, this shielding does not occur in the LTP configuration, causing ion loss before entrance to the mass analyzer. Nonetheless, the floating pin electrode creates a shielded environment similar to the DBDI configuration, increasing the number of ions reaching the mass analyzer. Understanding this phenomenon led to the development of the inverse‐voltage LTP (iLTP) ion source, where HV is applied to the needle electrode and the external annular electrode is grounded (Brandt et al. 2018). An optical emission study revealed that in the iLTP configuration, the plasma excitation wave travels from the tip electrode in a focused manner over long distances through the capillary, unlike in the DBDI or LTP configurations, where the excitation wave disperses at the position of the outer electrode. Additionally, it was found that the iLTP configuration can operate without a physical ground electrode (Klute, Brandt, and Franzke 2021). This resulted in the development of the flexible‐microtube plasma (FµTP) ion source, which benefits from having a particularly long plasma channel. This long channel enables the pin electrode to be retracted significantly far from the orifice of the dielectric capillary, thereby keeping it well away from the transfer capillary, preventing any potential short connection. Additional advantages of the FµTP device are its extremely small footprint and minimal gas flow, which is well below 100 mL/min (Brandt et al. 2018).

### 2.2 Vapor Introduction DBDI Sources

While DBDI has applications for analyzing liquids and solid surfaces, often in the so‐called ambient arrangement, as will be mentioned in Section 3, we focus this review on gaseous samples analyzed in closed configurations of flow systems. The class of ion sources dedicated to this is called vapor‐introduction ionization sources (Guo et al. 2015b). Liquids and solids need to be vaporized before they are introduced into such sources; several ways of doing this are overviewed in this section. Generally, the sample gas can either pass directly through the discharge or be added to the plasma after the discharge. We define “discharge gas” as gas from which the active plasma is created in the discharge. The term “carrier gas” refers to gas (usually containing the sample) in which reactions between energetic plasma species, the matrix, and analyte molecules occur. In some configurations, the discharge gas differs from the carrier gas, while in others, they are the same.

#### 2.2.1 Double Cylindrical DBDI

**Figure 2:**
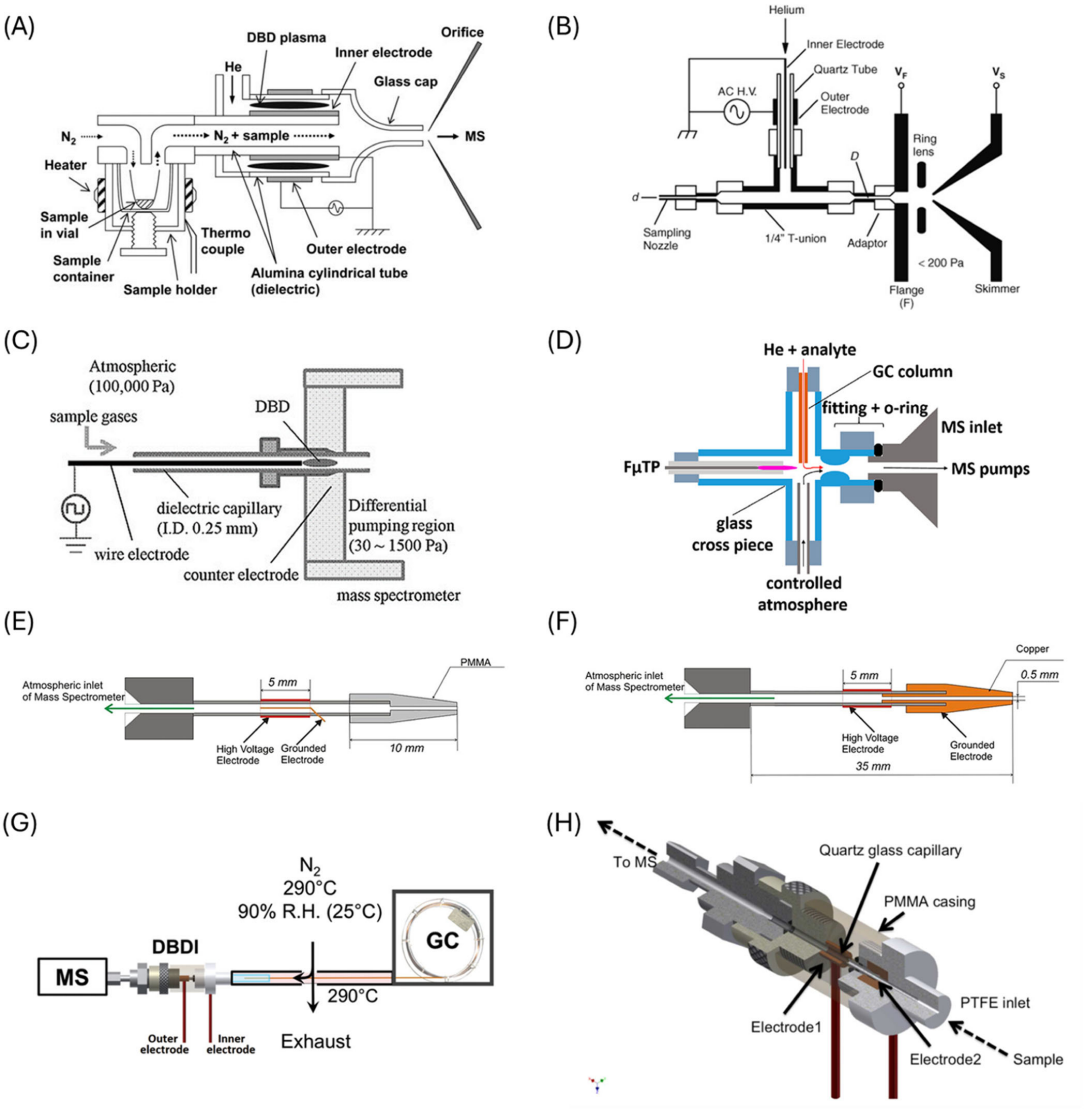
(A) Double cylindrical DBDI. Used with permission of Royal Society of Chemistry, from Hiraoka et al. (2011). Copyright 2011 The Royal Society of Chemistry. Permission conveyed through Copyright Clearance Center Inc. (B) Ambient sampling chemi/chemical ionization source. Adapted with permission from Chen et al. (2010a). Copyright 2010 John Wiley & Sons, Ltd. (C) LP‐DBDI Sugiyama et al. (2013). (D) CA‐FμTP. Adapted from Wang et al. (2020) licensed under CC‐BY‐NC‐ND 4.0. Copyright © 2020 American Chemical Society. (E) ACaPI in wire electrode configuration. Adapted with permission from Nudnova et al. (2012). Copyright 2012 John Wiley & Sons Ltd. (F) ACaPI in cap electrode configuration Adapted with permission from Nudnova et al. (2012). Copyright 2012 John Wiley & Sons Ltd. (G) ACaPI coupled to GC. Adapted with permission from Mirabelli, Wolf, and Zenobi (2017). Copyright 2016 American Chemical Society. (H) Schematic drawing of ACaPI source. Adapted with permission from Wolf et al. (2015). Copyright 2015 American Chemical Society. [Color figure can be viewed at wileyonlinelibrary.com]

In this configuration, DBD discharge through He is confined in the double cylindrical discharge tube so that the sample gas is not directly exposed to the He plasma (Hiraoka et al. 2011) (Figure 2A). Vaporized sample gases are carried by the N_2_ gas (200 mL/min) and transported to the exit of the dielectric tube, where the DBD excited He gas ionizes the gaseous sample molecules (see Section 4). The He flow rate is typically 1000 mL/min, and HV of several kV is used. The advantage of this ion source is that the ionization region is free from ambient air contaminants, resulting in high reproducibility. The analyte molecules are ionized without being exposed to active discharge, which results in the soft ionization of the gas sample. In one study, an LOD of a few picograms per mL was achieved for methamphetamine in an aqueous solution (Hiraoka et al. 2011).

#### 2.2.2 Ambient Sampling Chemi/Chemical Ionization Source

A schematic of one bespoke construction of ambient sampling low‐pressure chemi/chemical ionization source is shown in Figure 2B (Chen et al. 2010a). In this setup, metastable He* atoms are generated by DBD in the LTP configuration (platinum wire as the inner electrode and a copper strip as the outer electrode adhered to the outer surface of the quartz tube). He* metastables react with the sample gas in the junction of a stainless‐steel T‐union. The mixture of gases and ions is transferred into the mass spectrometer through an adapter and a custom‐made flange. The T‐fitting, where the ionization starts, is on the same electric potential as the flange. The inner electrode is held on the ground while 20 kHz AC HV (2000 V peak‐to‐peak) is applied on the outer one. For the measurement of the operating pressure of the ion source, the T‐union was replaced by a four‐way cross‐union, and the additional channel was connected with the pressure gauge. The operating pressure was about 90 mbar with the typical He flow rate of 80 mL/min. The pressure in the region between the flange and the skimmer was even lower at about 2 mbar. For the non‐volatile sample, heated nitrogen was used as a carrier gas, and an LOD of 1 ppbv for methamphetamine in solution and of 5 pg for solid hexamethylene triperoxide diamine was reported (Chen et al. 2010a). For the detection of gaseous hydrogen peroxide in ambient air, a detection limit of 0.8 ppbv was reported using a slightly different configuration of the DBD He^M^ source (the stainless steel connector at the end of the quartz tube was used as the inner electrode) (Chen, Yu, and Hiraoka 2010b).

#### 2.2.3 Low‐Pressure Dielectric‐Barrier‐Discharge Ion Source (LP‐DBDI)

Another configuration of DBDI called a low‐pressure dielectric‐barrier‐discharge ion source (LP‐DBDI), is shown in Figure 2C. This source consists of a dielectric capillary with the wire electrode inserted and the counter electrode attached around the dielectric capillary. A square‐wave voltage (2 kVp‐p, 1.5 kHz) was applied to the wire electrode, and the counter electrode was grounded. Vaporized samples are introduced from the ambient atmosphere into the capillary, and thus, ambient air is used as the discharge gas at a pressure of about 200 mbar. The samples pass directly through the DBD and its plasma jet. The outlet of the LP‐DBDI is connected to the vacuum region of the mass spectrometer, leading to high transmission efficiency for the generated ions. The pressure in the differentially pumped region (0.3–15 mbar) was regulated by introducing additional air into this region by a mass flow controller. Note that the DBD pressure was nearly independent of the pressure in the differential pumping region. It was observed that the relative intensities of the ions and the ionization efficiency were dependent on this pressure, indicating that the observable ions were generated in the differential pumped region (Sugiyama et al. 2013). The authors do not discuss possible diffusion losses explicitly.

#### 2.2.4 Controlled‐Atmosphere DBDI Sources

A DBDI source using a setup called *controlled atmosphere flexible microtube plasma (CA‐FμTP)* (Figure 2D) was shown to significantly increase sensitivity and improve LODs. The controlled atmosphere (pure N_2_/O_2_ mixture) significantly reduced the chemical noise in the mass spectrometer. Moreover, tailoring the atmosphere for a specific class of analytes allowed control of the chemical reaction pathways, thereby optimizing the ionization process for the desired outcomes (Vogel et al. 2019).

This source was coupled to the GC column to analyze separated eluent. The FμTP capillary has a 250 μm inner diameter and contains a 100 μm tungsten wire as the HV electrode ending 10 mm before the fused silica outlet. The helium flow rate through the FµTP is 100 sccm; the controlled atmosphere (CA: 80% N_2_, 20% O_2_) flow rate is 500 sccm. Rectangular AC HV 2.0 kV peak‐to‐peak, 20 kHz was used. Pressure at the point where the plasma interacts with the sample and CA determined from the fluid dynamics simulations is 2.5 mbar. The composition of CA was found to give optimal signals of NO^+^ and H_5_O_2_^+^ for 20% of O_2_ in the mixture. The geometry of the glass cross was optimized to a special constricted shape that enhanced the reproducibility of the measurements. Within multiple measurements over several months of a 100 ppbv mixture of trace VOC vapors, the variation of signal intensities was below 6%. An LOD for volatile organic compounds found in human saliva was 80 ppt (Wang et al. 2020).

Another design of a controlled atmosphere ion source coupled to a GC was the iLTP ion source, which was inserted into a custom‐made housing similar to the commercial GC‐APCI source of the MS (Ayala‐Cabrera et al. 2022). The ionization behavior was evaluated using an analytical standard mix of compounds. Testing with He and Ar discharge gases revealed that Ar provided signal responses twice as high as He. Since Ar^M^ does not have sufficient energy to ionize N_2_ by Penning ionization, a process dominant in He plasma, the roles of Ar_2_^+^ and VUV emission of Ar* states were considered to explain the ionization of water molecules or analytes directly or water. The addition of water vapor directly into the source was also tested, showing that most analytes had increased responses under wet conditions. The impact of different auxiliary gases (N_2_, O_2_, synthetic air) on the ionization response was also evaluated. The use of N_2_ increased ionization efficiency for most analytes, while neat O_2_ generally led to the lowest responses, except for certain compounds. This observation was explained by the possible role of O_2_^−^ anions in reducing the signal of positive ions. Interestingly, compounds prone to generating molecular ions, such as highly halogenated compounds and those with multiple NO_2_ groups, showed higher responses when O_2_ was used as the auxiliary gas (Ayala‐Cabrera et al. 2022).

#### 2.2.5 Active Capillary Plasma Ionization (ACaPI) Source

The active capillary plasma ionization (ACaPI) source is based on a quartz tube (1 mm inner diameter) that serves as both the dielectric barrier and the sampling capillary (Nudnova et al. 2012). The ionization takes place within a confined volume inside the capillary. The “wired electrode” design (Figure 2E) represents an LTP configuration; the drawback of this configuration is that the wire disturbs the airflow and can adsorb analyte molecules. This is avoided in the “cap electrode” design shown in Figure 2F. Ambient air with an analyte is transported by convection through the DBD into the transfer line of the mass analyzer. The gas flow through the source is typically 1500 mL/min. The discharge is driven by 2.5–5 kV, 10 kHz AC HV. Testing with traces of decylamine standard showed that the “cap electrode” design is about ten times more sensitive than the “wire electrode” design. Also, it was found that the cap electrode requires half the power of the wire electrode to start the discharge, and the volume of plasma in the cap electrode's active capillary is much larger compared to the wire electrode configuration. The generator's power does not significantly affect the analyte's ionization efficiency, but it results in a higher signal‐to‐background ratio for both configurations of the active capillary when the power delivered to the plasma is lower (Nudnova et al. 2012). It was shown that when the active capillary source is used with pure nitrogen as discharge gas, an increase of the HV from 2.4 kV to 3.4 kV allows the identification of alkylbenzene isomers based on N atom adducts (Begley and Zenobi 2023). Methods based on thermometer ions indicated that ions generated by ACaPI have lower internal energy compared to those generated by APCI, DART, LTP, and FµTP sources and slightly higher energy than the ions generated by ESI sources (Stephens et al. 2015; Dumlao et al. 2017a; Bouza et al. 2023a). Using sub‐microsecond HV pulses to drive ACaPI can further increase sensitivity, signal‐to‐noise ratios, and LODs (Ahmed et al. 2020).

This ACaPI was also coupled to GC, and its performance was demonstrated for soft ionization of separated pesticides and illicit drugs (Figure 2G). Humidified N_2_ (90% R.H. at 25°C) was used as a discharge gas. Humidification was pointed out as an important step in increasing ionization efficiency by proton transfer from hydronium clusters, leading to the formation of MH^+^ ions with minimal fragmentation. This is because the molecules are primarily ionized by secondary reaction processes that occur after the plasma region, where neutral molecules interact with the ions generated in the plasma. The typical flow of He used in GC was ~3 mL/min, and the total nitrogen flow entering the ionization source was ~1500 mL/min. It was shown that He is not involved in ionization as increasing its flow up to 20 mL/min has no noticeable effect on the recorded mass spectra (Mirabelli, Wolf, and Zenobi 2017).

A direct coupling of solid phase microextraction (SPME) with ACaPI was reported in 2016 by Renato Zenobi's group (Mirabelli, Wolf, and Zenobi 2016). Another interesting approach with SPME fiber acting as a discharge electrode (Dumlao et al. 2016) was used to analyze chemical warfare agent simulants.

### 2.3 Commercially Available DBDI Sources

The DBDI method has been implemented in commercial devices designed as add‐ons to standard mass analyzers providing high sensitivity with minimal sample preparation. Two products are mentioned in the current scientific literature.

#### 2.3.1 DBDI‐100 by China Innovation Instrument

One of the first commercially available implementations of DBDI was an ion source developed by Huayi Ningchuang, which is supplied by China Innovation Instrument Co. Ltd, (cii‐tech. com 2024) a company producing mass spectrometers, pre‐processing equipment, reagents, mobile laboratories, and related software. This development stemmed from the research of DBDI by Xinrong Zhang and colleagues at Tsinghua University in 2007 (Na et al. 2007). In the year 2016, the China Innovation Instrument produced a patented DBDI‐100 ion source (Zhao and Wen 2015). Its notable features include single‐electrode discharge technology, which allows for an external ejection length of the ion beam source greater than 4.5 cm, enhancing the feasibility of on‐site, in‐situ analysis and Vacuum‐Assisted Ionization, reducing background noise and high temperature. The carrier gas can be heated to temperatures up to over 400°C at flow rates 0.2–5.0 L/min, supporting simultaneous mixing of multiple gases. The innovative features also include high temperature and high voltage safety assurance technology and elimination of signal crosstalk.

DBDI‐100 can be used in conjunction with mass analyzers from mainstream mass spectrometry manufacturers to achieve direct sample injection and analysis of gas, liquid, and solid samples and is suitable for in‐situ, real‐time, and rapid detection in the fields of food safety, drug safety, drug inspection, environmental monitoring, clinical diagnosis, public safety, and chemical research.

The scientific studies realized using this ion source include identification perfluorinated carboxylic acids in real textile samples, (Wang et al. 2020) chemical warfare agent simulants in soil (Li et al. 2020) and fentanyl analogs in plasma and blood spot samples (Li et al. 2022). Coupling of DBDI‐100 to capillary electrophoresis was demonstrated on analyses of common pharmaceuticals metronidazole and acetaminophen (Zhang et al. 2016).

#### 2.3.2 SICRIT by Plasmion

The optimization of the Active Capillary Plasma Ionization (ACaPI) source (Nudnova et al. 2012) resulted in the development of the commercially available SICRIT (an acronym standing for “Soft Ionization by Chemical Reaction In Transfer”) patented ion source by Plasmion GmbH in the year 2016 (Plasmion. com 2024). This source operates on principles stemming from fundamental research on ACaPI (Wolf et al. 2015; Mirabelli, Wolf, and Zenobi Wolf, and Zenobi 2016, 2017). Figure 2H shows schematics of the SICRIT's predecessor (Wolf et al. 2015) (note that the schematics of the commercial device are proprietary).

Plasmion offers dedicated coupling of the SICRIT ion source to all commercial mass spectrometry (MS), gas chromatography‐mass spectrometry (GC‐MS), and liquid chromatography‐mass spectrometry (LC‐MS) systems, including a heated sampling line module. The supplied generator provides a 0–3000 Vpp AC sinusoidal voltage within a frequency range of 10–50 kHz, ensuring a wide operational range and compatibility with various analytical setups.

The SICRIT ion source is based on a patented cold plasma technique and currently represents one of the most promising commercial implementations of the DBDI principle. SICRIT extends the MS inlet and ionizes molecules in the sample using a uniquely shaped cold plasma DBD discharge immediately before they are drawn into the transfer line. This approach reduces sample loss due to charge repulsion, increasing sensitivity and ion yield. Installation and operation are straightforward, requiring no calibration or significant adaptations to existing hardware or software. The system operates using ambient air and electrical power, eliminating the need for costly noble gases like helium.

SICRIT enables direct, quantitative MS analysis of solid, liquid, or gaseous samples without the need for prior chromatography. It is compatible with LC‐MS systems from various vendors and can be seamlessly integrated with GC, LC, and SFC methods. This flexibility allows for combining the high separation power from chromatography with the soft ionization and sensitivity of LC‐MS, providing cleaner spectra with fewer adducts.

The scientific studies realized using the SICRIT ion source include the identification of characteristic VOCs of *Aspergillus* sp., *Ceratocystis* sp., and *Neurospora* sp. filamentous fungi (Heffernan et al. 2023), monofloral honey (Massaro et al. 2024), yak milk (Zhang et al. 2024) and essential oils (Raeber and Steuer 2023). SICRIT was also demonstrated for analyses of polycyclic aromatic hydrocarbons (Huba, Mirabelli, and Zenobi 2019) and polar trace contaminants in water matrices (Huba, Mirabelli, and Zenobi 2018), and of illicit drugs extracted from fingerprints (Conway et al. 2023). An infrared‐radiation‐based evaporation system with a SICRIT source was able to identify polycyclic aromatic hydrocarbons in vehicle engine exhaust aerosol and observe changes in composition during the engine test cycle (Thaler et al. 2021). The SICRIT ion source was also used in combination with GC to analyze saturated aliphatic hydrocarbons in diesel fuel (Weber et al. 2021).

## 3 Ion Chemistry

In a dielectric barrier discharge (DBD), the plasma is sustained by a continuous supply of energy from an electric field. This electric field accelerates free electrons to kinetic energies high enough to ionize neutral gas, creating secondary electrons and positive ions. The collisions between electrons and atoms or molecules of the discharge gas also form electronically excited atoms or molecules and the dissociation of molecules into atoms, such as N in nitrogen (Begley and Zenobi 2023) or O in air. These atoms, in turn, can react with the gas molecules forming, for example, nitrous oxide (Herron and Green 2001):

(1) $$N^{∙}+O_{2}\to {\text{NO}}^{∙}+O$$

Similarly, O atoms can form ozone O_3_ (Siemens 1857), and other oxides of nitrogen can also be generated in subsequent reactions. These molecules can thus affect the ionization process, even if they are not directly evident on the mass spectra.

When the discharge gas is He or Ar, metastable excited neutral atoms are important species for the ionization of the analyte molecules by Penning ionization.

(2) $${\text{He}}^{*}+M\to M^{+∙}+e^{-}$$

The rate coefficients for Penning ionization reactions are typically 10^−11^ cm^3^s^−1^. This means the He* metastable atoms can survive in relatively pure He gas even in the presence of 1 part per million by volume (ppmv) impurity of H_2_O (see Table 1) for more than a millisecond. However, they will react with N_2_ gas at near atmospheric pressure in less than a microsecond forming N_2_^+⋅^ (Speicher et al. 2024). Note that other rare gas discharges were also studied, showing that N_2_^+⋅^ acted as reagent ions in this situation (Tian et al. 2024). It is important to note that there is no evidence for Penning ionization in nitrogen or air discharge gas.

**Table 1:** Key terms for reaction processes.

Gas number density	The concentration of gas atoms or molecules per unit volume, conventionally expressed in units of cm^−3^. Useful values are 2.7 × 10^19^ cm^−3^ at 1 atm and 0°C (Loschmidt number), 2.4 × 10^19^ cm^−3^ at 1 bar and 300 K. Thus, 1 ppmv admixture corresponds to 2.4 × 10^13^ molecules per cm^3.^
Penning ionization	An ionizing reaction in which an excited metastable atom or molecule transfers energy to a neutral molecule, resulting in the ejection of an electron
Ion‐molecule reaction	A chemical reaction where an ion interacts with a neutral molecule, resulting in the transfer of electrons or atoms, producing new ion species
Binary Ion‐molecule reaction	An ion‐molecule reaction, in which the excess energy is released in kinetic energy of the products
Ion‐molecule reaction rate coefficient	A probability of an ion reacting with a molecule per unit time and per unit concentration of the molecules. Conventionally expressed in cm^3 ^s^−1^ for binary reactions (1–4 × 10^−9^ cm^3 ^s^−1^) or in cm^6 ^s^−1^ for ternary reactions.
Ternary Ion‐molecule reaction	An ion‐molecule reaction, in which the excess energy is released in a collision with the molecule of the bath gas. Typically, an association reaction forms adduct ions.
Non‐dissociative Electron Attachment	A capture of a free electron by a neutral molecule without breaking any bonds, forming a stable negative ion, usually by a ternary process involving the bath gas molecules
Dissociative Electron Attachment	A reaction of a free electron with a molecule leads to the formation of two or more fragments, one of which carries a negative charge.

The primary ions formed in the discharge quickly react with neutral atoms and molecules by ion‐molecule reactions. These reactions can be binary (two‐body), where the excess energy is released as the kinetic energy of the products, or ternary (three‐body), where the excess energy is released in the collision of the reaction intermediate complex with the atom or molecule of the discharge gas. At atmospheric pressure in the DBD ion source, the three‐body reactions proceed very efficiently, and their effective rate coefficients, *k*, are equivalent to the collisional rate coefficients, *k*_c_, typically 10^−9^ cm^3^ s^−1^. Thus, the initially formed discharge gas cations will be rapidly associated with the neutral gas as:

(3) $${\text{He}}^{+∙}+2\text{He}\to {\text{He}}_{2}^{+∙}+\text{He}$$

(4) $$N_{2}^{+∙}+2N_{2}\to N_{4}^{+∙}+N_{2}$$

As the number density of the discharge gas is 2.7 × 10^19^ cm^−3^, this association will proceed on timescales much shorter than 1 µs. Thus, in a typical DBD ion source, where the residence time is more than 100 µs, it can be considered for any practical purposes that the initial reagent ions are of the type of N_4_^+⋅^. However, these ions are highly reactive due to their high recombination energy (14 eV) and will react by electron transfer with most impurities in the discharge gas. For example, the reaction of N_4_^+⋅^ with H_2_O (*k* = 3 × 10^−9^ cm^3^ s^−1^) (Smith, Adams, and Miller 1978) will proceed on a timescale of 10 µs when water is present at 1 part per million by volume, ppmv, of the atmospheric pressure gas. The details of the ion chemistry initiated by these positive ions will be discussed in a separate section later.

Free electrons, *e* ^–^, generated in the discharge can form negative ions by three‐body electron attachment or dissociative electron attachment. In practice, the initial negative ions can be O_2_^–⋅^ or O^–⋅^. Note that the rate coefficients for electron attachment to O_2_ are 3 × 10^−30^ cm^6^s^−1^ for three body attachments and are highly sensitive to electron energy for dissociative attachment, which is also slow. This means that at a 20% concentration of O_2_ in the air at 1 bar, the conversion of free electrons to O_2_^–⋅^ or O^–⋅^ takes place on a timescale of a fraction of a µs. However, at an O_2_ concentration of 1 ppmv, the free electrons can survive for an ms, long enough to ionize the sample directly.

It is also important to keep in mind that the composition of ions in the source at atmospheric pressure does not directly correspond to their relative intensities on the mass spectra. This may be in part due to the diffusion losses in the transfer capillary, which can be significant, especially for small ions (in the sense of geometrical size); thus, for example, the signal of O^–⋅^ can be much smaller than what would correspond to the concentration in the source. Also, the heating of the transfer line and the ion optics involved in the transfer of the ions to the high vacuum of the mass analyzer are normally optimized to dehydrate hydrated ions by collisions with the carrier gas molecules.

### 3.1 Positive Ion Mode

**Figure 3:**
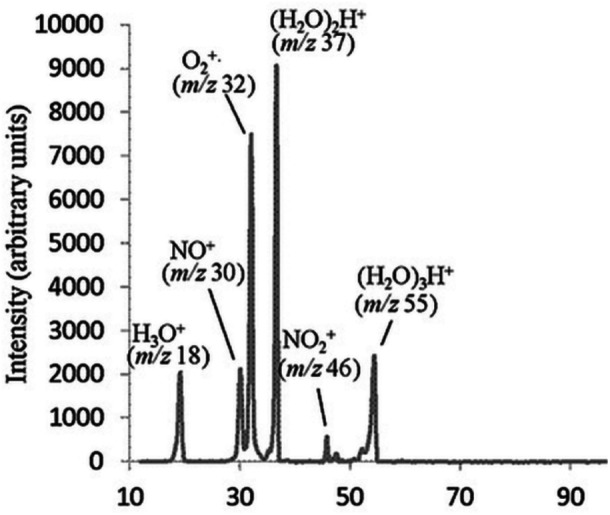
Reagent ions produced by a low‐pressure DBDI source with air as discharge gas. Adapted with permission from Sugiyama et al. (2013). Copyright 2013 John Wiley & Sons Ltd.

The chemistry of discharges at atmospheric or near‐atmospheric pressures is well understood. In the context of mass spectrometry, historically, the focus was on atmospheric pressure chemical ionization, APCI. The ionization and reaction mechanisms in DBD‐based ion sources are similar to those in APCI (Wolf et al. 2016; Bouza et al. 2023b). The main reagent ions in discharge through humid air are H_3_O^+^(H_2_O)_0,1,2…_, NO^+^, and O_2_^+⋅^, as illustrated by a mass spectrum obtained from a low‐pressure DBDI source shown in Figure 3 (Sugiyama et al. 2013). The following sections give an overview of processes involved in the formation of reagent ions in different discharge gases and a summary of their reactions with analyte molecules leading to characteristic ions observed on the mass spectra.

#### 3.1.1 Reagent Cations

##### 3.1.1.1 Helium Discharge Gas

Helium is a gas often used in plasma sources. The primary process in the helium plasma is the interaction of energetic electrons with helium atoms, during which the He^+⋅^ ions are formed by reactions ((5a), (5b), (5c)) (Martens et al. 2009), inelastic collision traditionally denoted as electron impact and by metastable‐metastable ionization process (6) (Deloche et al. 1976):

(5a) $$\text{He}+e^{-}\to {\text{He}}^{*}+e^{-}$$

(5b) $$\text{He}+e^{-}\to {\text{He}}^{+∙}+2e^{-}$$

(5c) $${\text{He}}^{*}+e^{-}\to {\text{He}}^{+∙}+2e^{-}$$

(6) $${\text{He}}^{m}+{\text{He}}^{m}\to {\text{He}}^{+∙}+\text{He}+e^{-},k=1.5\times 10^{-9}\;{\text{cm}}^{3}s^{-1}$$

At higher pressures, the population of He_2_^+⋅^ dominates over He^+⋅^. There are at least three ways for He_2_^+⋅^ formation. The most important is association (ion conversion) reaction (Phelps & Brown 1952; Bohringer, Glebe, and Arnold 1983; Golubovskii et al. 2003):

(7) $${\text{He}}^{+∙}+2\text{He}\to {\text{He}}_{2}^{+∙}+\text{He},k=1.1\text{to}1.4\times 10^{-31}{\text{cm}}^{6}s^{-1}$$

The excess energy is released by collisions with He atoms as third bodies (McDaniel et al. 1970). This ternary association reaction (7) is very efficient; from its rate coefficient, it follows that the time for conversion of He^+⋅^ to He_2_^+⋅^ is 12 ns at atmospheric pressure (Stevefelt et al. 1982). Modeling of a dielectric‐barrier discharge showed that He_2_^+⋅^ completely dominates the positive charge density at pressures higher than 200 mbar (Martens et al. 2007).

Other possible routes are metastable–metastable ionization (Deloche et al. 1976; Bohringer, Glebe, and Arnold 1983)

(8) $${\text{He}}^{m}+{\text{He}}^{m}\to {\text{He}}_{2}^{+∙}+e^{-},k=1.5\text{to}2.0\times 10^{-9}{\text{cm}}^{3}s^{-1}$$

and associative ionization by highly excited He atoms (Hornbeck and Molnar 1951; Alves, Gousset, and Ferreira 1992)

(9) $${\text{He}}^{*}\left(n\geq 3\right)+\text{He}\to {\text{He}}_{2}^{+∙}+e^{-},k=0.13\text{to}8.2\times 10^{-10}{\text{cm}}^{3}s^{-1}$$

Whatever the details of these processes, it can be assumed that at atmospheric pressure, all He^+⋅^ ions are converted to He_2_^+^ almost immediately.

##### 3.1.1.2 Nitrogen Discharge Gas

When nitrogen is used as a discharge gas, the following reactions occur. In pure nitrogen discharge, N_2_^+⋅^ ions are formed in collisions with energetic electrons.

(10) $$N_{2}+e^{-}\to N_{2}^{+∙}+2e^{-}$$

At sufficiently high pressures, the N_2_^+⋅^ ions undergo ternary association reaction (Good et al. 1970a; Dzidic et al. 1976)

(11) $$N_{2}^{+∙}+2N_{2}\to N_{4}^{+∙}+N_{2},k=8\times 10^{-29}{\text{cm}}^{6}s^{-1}$$

The high rate coefficient of this reaction ensures that even at a relatively low pressure of 3.5 mbar, N_4_^+^ ions become dominant ionic species in ~20 µs after irradiation with a 10 µs electron pulse (Good et al. 1970a). Besides N_4_^+⋅^ ions, N_3_^+^ ions were also observed at the same conditions (Good et al. 1970a). This ion is formed by the ternary association reaction of the N^+^ ion (Good et al. 1970a; Freysinger et al. 1994):

(12) $$N^{+}+2N_{2}\to N_{3}^{+}+N_{2},k=5\times 10^{-29}{\text{cm}}^{6}s^{-1}$$

Appearance energy measurements revealed that N_3_^+^ can also be formed by the reaction of excited states of N_2_^+⋅^ with N_2_ (Stephan et al. 1984):

(13) $$N_{2}^{+∙*}+N_{2}\to N_{3}^{+}+N^{∙}$$

The N^+^ ions entering the reaction (12) are mostly created by the dissociative ionization of nitrogen molecules,

(14) $$N_{2}+e^{-}\to N^{+}+N^{∙}+2e^{-}$$

that can, at higher electron energy, occur in parallel with the reaction (8). N^+^ can also stem from other minor reaction channels (Tsonev et al. 2023).

In the presence of helium (e.g., in the afterglow of the He‐LTP), N_2_^+⋅^ could also be formed by various reactions with high‐energy helium species (He*, He_2_*, He^+⋅^, He_2_^+⋅^) such as electron transfer reactions (15) and (16), and Penning ionization reaction (17):

(15) $${\text{He}}_{2}^{+∙}+N_{2}\to N_{2}^{+∙}+2\text{He}$$

(16) $${\text{He}}^{+∙}+N_{2}\to N_{2}^{+∙}+\text{He}$$

(17) $${\text{He}}^{m}+N_{2}\to N_{2}^{+∙}+\text{He}+e^{-}$$

The N_2_^+⋅^ ions thus formed will again rapidly associate with N_2_ in reaction (11). Thus, in practical DBDI arrangements, N_4_^+⋅^ ions are the key reagent ions for subsequent ionization of other components.

##### 3.1.1.3 Presence of Water Vapor

Ionization energy of H_2_O (12.6 eV) is lower than that of He (24 eV) or N_2_ (15.6 eV), and thus, even a small amount of water vapor impurity in discharge gas will lead to the formation of water ions.

This was already observed in 1965 by Shahin in a mass spectrometric study of corona discharge in ambient air at atmospheric pressure using a quadrupole mass spectrometer with a differentially pumped discharge tube (Shahin 1966). The major influence of the presence of water vapor in ionized air was manifested by the dominant presence of H_3_O^+^(H_2_O)_*n*_ ions at water concentrations as low as 410 ppmv. This ion chemistry is well known and has been observed many times because of its importance for the atmosphere. One notable example is a study by Kebarle and Hogg, who irradiated mixtures of gases and water by *α*‐particles (Kebarle and Hogg 1965b, 1965a). The following reactions are most important for the formation of H_3_O^+^ ions in N_2_ containing H_2_O at the atmospheric pressure (Shahin 1966)

(18) $$N_{4}^{+∙}+H_{2}O\to 2N_{2}+H_{2}O^{+∙},k=1.8\times 10^{-9}{\text{cm}}^{3}s^{-1}$$

(19) H2O+∙+H2O→H3O++OH∙,k=1.9×10−9cm3s−1

Good et al. (1970a) studied ion‐molecule reactions in pure and moist nitrogen at pressures of 0.7–5.3 mbar and measured the kinetics of clustering reactions forming H_3_O^+^(H_2_O)_*n*_. They concluded that the formation of protonated water proceeds almost exclusively by reactions (18) and (19), emphasizing the importance of N_4_^+⋅^ ions in the nitrogen discharge plasma at higher pressures (vide supra).

Whilst the H_3_O^+^ hydronium ions are relatively unreactive with the main components of air, they do slowly associate with H_2_O to form hydrated H_3_O^+^(H_2_O)_n_ ions. The kinetics of this process in N_2_ containing traces of H_2_O was studied in the nitrogen pressure range of 1.6–4.3 mbar at water vapor partial pressures 4 × 10^−4^–9.3 × 10^−3^ mbar (Good et al. 1970a) The ternary rate coefficients for reactions (20)–(22) were measured (Good et al. 1970a)

(20) $$H_{3}O^{+}+H_{2}O+N_{2}\to H_{3}O^{+}H_{2}O+N_{2},k=3.4\times 10^{-27}{\text{cm}}^{6}s^{-1}$$

(21) $$H_{3}O^{+}H_{2}O+H_{2}O+N_{2}\to H_{3}O^{+}{\left(H_{2}O\right)}_{2}+N_{2},k=2.3\times 10^{-27}{\text{cm}}^{6}s^{-1}$$

(22) $$H_{3}O^{+}{\left(H_{2}O\right)}_{2}+H_{2}O+N_{2}\to H_{3}O^{+}{\left(H_{2}O\right)}_{3}+N_{2},k=2.4\times 10^{-27}{\text{cm}}^{6}s^{-1}$$

This sequence can continue indefinitely, but depending on temperature, the reverse collisional dissociation reactions will lead to the thermodynamic equilibrium distribution of H_3_O^+^(H_2_O)_*n*_ with a maximum around *n* = 5 (Dryahina et al. 2021a).

##### 3.1.1.4 Presence of Oxygen

The reaction of N_3_^+^ and N_4_^+⋅^ with O_2_ was studied by Dunkin et al. using the flowing afterglow technique (Dunkin et al. 1971) Three products of these reactions were found, namely, O_2_^+⋅^, NO^+^, and NO_2_^+^, stemming from the following reactions:

(23) $$N_{4}^{+∙}+O_{2}\to O_{2}^{+∙}+2N_{2}$$

(24) $$\begin{array}{ccc} N_{3}^{+}+O_{2} & \to & {\text{NO}}^{+}+O+N_{2}\\ \; & \to & O_{2}^{+}+N^{∙}+N_{2}\\ \; & \to & {\text{NO}}_{2}^{+}+N_{2}\; \end{array}$$

These product ions are well established. Already, Shahin observed NO^+^ and NO_2_^+^ in corona discharge in dry and moist air (Shahin 1966) and O_2_^+⋅^ in corona discharge in a mixture of nitrogen and oxygen (Shahin 1966). Dzidic et al. (1976) observed these ions in atmospheric pressure ionization using ^63^Ni and corona discharge in nitrogen‐containing a trace amount of oxygen. Siegel and Fite (1976) observed O_2_^+⋅^, NO^+^, and NO_2_^+^ and their hydrates in ^63^Ni atmospheric pressure ionization of nitrogen, argon, and air and discussed their origin and possible utilization for trace impurity analysis in gases. Similar observations were also made by Kambara et al. (1979), who utilized collision dissociation as a tool for the identification of various isobaric cluster ions produced in an atmospheric pressure ionization process.

Of course, when air is used as discharge gas, O_2_ can be ionized directly by energetic free electrons.

(25) $$O_{2}+e^{-}\to O_{2}^{+∙}+2e^{-}$$

All ions resulting from the presence of O_2_ are, similarly like H_3_O^+^, unreactive with major components of air, but they slowly associate with H_2_O in ternary reactions (Good et al. 1970b; Fehsenfeld et al. 1971a) and ultimately switching reactions convert their hydrates to H_3_O^+^(H_2_O)_n_ (Smith and Spanel 1995; Španěl & Smith 2009):

(26) $${\text{NO}}^{+}{\left(H_{2}O\right)}_{3}+H_{2}O\to H_{3}O^{+}{\left(H_{2}O\right)}_{2}+{\text{HNO}}_{2}$$

(27) $$O_{2}^{+∙}{\left(H_{2}O\right)}_{2}\to H_{4}O_{2}^{+∙}+O_{2}$$

(28) H4O2+∙+H2O→H3O+H2O+OH∙

This means that in humidified air, the reagent ions in DBDI will be largely H_3_O^+^(H_2_O)_*n*_ (Fehsenfeld and Ferguson 1969; Fehsenfeld et al. 1971b).

##### 3.1.1.5 Presence of Ammonia

The influence of ammonia in the discharge gas was mentioned by Shahin (1966) and Dzidic et al. (1976).

Besides the direct ionization by the energetic plasma electrons, the NH_3_^+^^⋅^ ion can be formed by electron transfer from NH_3_ to any of the energetic ions in the discharge plasma, that is, He^+^^⋅^, He_2_^+^^⋅^, N^+^, N_2_^+^^⋅^, N_4_^+^^⋅^, etc. Due to the high exothermicity of these electron transfer reactions, NH_3_^+^^⋅^ ions are assumed to be formed together with their fragments NH_2_^+^ and ^⋅^NH^+^^⋅^ (Dzidic et al. 1976) Depending on the ammonia concentration and the presence of water in the discharge gas, the ammonium ion NH_4_^+^ can be formed.

(29) NH3+∙+NH3→NH4++N∙H2

(30) $$H_{3}O^{+}+{\text{NH}}_{3}\to {\text{NH}}_{4}^{+}+H_{2}O$$

Ammonium ions can be solvated by ammonia and water molecules (Hogg and Kebarle 1965).

#### 3.1.2 Ionization of Analyte Molecules

The metastable electronically excited atoms and reagent ions react with the analyte molecules by a variety of mechanisms, that will be summarized in this section. The product ions of these reactions can then further associate with H_2_O or dopant molecules and possibly even with other analyte molecules.

##### 3.1.2.1 Penning Ionization

This mechanism usually takes place in DBDI operating with He discharge gas, but in principle, it can also occur in Ar discharge gas. The reaction (2) leads to the formation of radical cation M^+^ (Bouza et al. 2023b) He* metastables (19.8 eV) will react with all common molecules as their ionization energies are below 14 eV. Ar* metastables (11.7 eV) are less energetic but still can react with most organic molecules except acetonitrile and methane. Penning ionization does not take place in N_2_ or air discharge gases.

##### 3.1.2.2 Electron Transfer

When radical cations formed from the discharge gas live long enough to react with analyte molecules before they are converted to protonated close shell cations, electron transfer (also called charge transfer) can possibly take place:

(31a) $$A^{+}+M\to A+M^{+}$$

(31b) $$\to A+X+{\left(M-X\right)}^{+}$$

The primary ionizing species A^+^ depends on the type of discharge gas being utilized. In an atmospheric pressure discharge, it is mainly He_2_^+^^⋅^, while in nitrogen, it is N_4_^+^^⋅^. The degree of fragmentation indicated by the reaction channel (31b) depends on the difference between the ionization energies of A and M and on the chemical stability of M. Considering nitrogen, the recombination energy of N_4_^+⋅^ (14.6 eV) is about 1 eV lower than that of N_2_^+⋅^ (15.6 eV). As the ionization energies of most organic molecules range between 7 and 11 eV, the electron transfer to N_4_^+⋅^ will be exothermic by more than 3 eV, an amount of energy sufficient to rearrange the structure of M^+⋅^ and break bonds, leading to fragmentation (31b). Interestingly, this energy is similar to the energy deposited to analyte molecules by electron ionization with 70 eV electrons, and thus, the fragments observed in nitrogen discharge gas DBDI would be a subset of those present in EI spectra. It is important to note that this ionization mechanism in DBDI is only feasible for extremely dry discharge gas. Currently reported studies in the literature do not provide any examples of this mechanism.

##### 3.1.2.3 Proton Transfer

When the discharge gas contains a small amount of water vapor, the reagent ions include H_3_O^+^ or possibly NH_4_^+^ in the presence of ammonia. These ions react with the analyte molecules M by proton transfer when their proton affinity is sufficient:

(32a) $${\text{AH}}^{+}+M\to {\text{MH}}^{+}+A$$

(32b) $$\to {\left(\text{MH}-X\right)}^{+}+X+A$$

where A is usually H_2_O and sometimes NH_3_, as explained in the previous section. Reaction (32a) is a non‐dissociative proton transfer that takes place when chemically stable protonated molecules are formed (as is the case for aromatic and unsaturated hydrocarbons) at *m/z* 1 unit above the molecular mass of M. Dissociative proton transfer occurs for many classes of organic molecules: protonated alcohols and aldehydes tend to lose X = H_2_O molecules, forming product ions at *m/z* 17 units below molecular weight of M. Other common fragments are *m/z* 81 for monoterpenes and those corresponding to a loss of OR alkoxy radicals for protonated ethers and esters. The chemistry of gas‐phase proton transfer reactions is well understood due to its importance for PTR‐MS and SIFT‐MS (Smith, McEwan, and Španěl 2020). Thus, in positive mode DBDI, the proton transfer is a commonly seen ionization mechanism for organic molecules that leads to MH^+^ (Zhang et al. 2024). It should be noted that many alkanes do not readily protonate, and instead, they form oxidized ions of the type [M − nH + mO]^+^ in DBDI (Weber et al. 2021) or lose an H atom to form [M − H]^+^ as observed in alkylbenzenes (Begley and Zenobi 2023). A word of caution needs to be made that while it is true that DBDI often leads to the formation of MH^+^ (Massaro et al. 2024), the reverse is not true; the appearance of an ion on a positive mass spectrum does not unequivocally indicate the existence of analyte molecules with a molecular weight one unit less than the observed *m/z* (Heffernan et al. 2023). A final note related to MH^+^ products is, that at larger analyte concentrations they can associate to form M_2_H^+^ secondary ions.

##### 3.1.2.4 Ligand Switching

As pointed out in the previous section, when the partial pressure of water vapor in discharge or carrier gas is sufficiently high, H_3_O^+^(H_2_O)_n_ hydrates become the dominant reagent ions. They will then react with analyte molecules by ligand‐switching reactions

(33) $$H_{3}O^{+}{\left(H_{2}O\right)}_{m+n}+M\to {\text{MH}}^{+}{\left(H_{2}O\right)}_{m}+(n+1)H_{2}O$$

forming hydrated protonated molecules. The reactivity of H_3_O^+^(H_2_O)_*m*+*n*_ with molecules is in general governed by thermochemistry and they proceed rapidly only when they are exothermic. When they are close to thermoneutral, the abundance of the MH^+^(H_2_O)_*m*_ product ions will be determined by thermodynamic equilibrium and can be related to concentrations of M and H_2_O considering the change in Gibs free energy. The general aspects of this chemistry are similar to SESI, see (Dryahina et al. 2021b; Som et al. 2021; Španěl et al. 2023). The main difference is due to reaction times and respective contributions of reactions taking place in the actual ion source and in the transfer line. It is important to note that whilst hydrated ions are formed in the atmospheric pressure region of the ion source, they are typically dehydrated by elevated temperatures in the transfer lines and in collisions with gas in the mass analyzer entrance ion optics. Thus, in the default configurations of commercial mass analyzers MH^+^ ions occur on the mass spectra.

### 3.2 Negative Ion Mode

**Figure 4:**
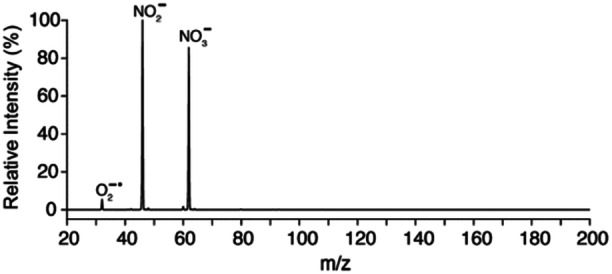
Mass spectrum of the background ions in negative polarity using air as the discharge gas. Adapted with permission from Gyr et al. (2018). Copyright 2018 American Chemical Society.

The negative ion chemistry in DBDI was recently well explained in an article on mechanistic understanding of DBDI by Gyr et al. (2018). It is thus well established that the main negative reagent ions formed in atmospheric pressure air discharge plasma are NO_3_^−^, NO_2_^−^, and O_2_^−⋅^ (see Figure 4).

The ion chemistry leading to their formation has been researched for several decades due to its importance for atmospheric chemistry and is thus understood in detail as summarized here.

#### 3.2.1 Reagent Anions

Primary negative ions in discharge plasma can be formed by interaction with electrons in three different ways:

**Table:** 

1. Ternary electron attachment	X + *e* ^−^ + M ⟶ X^−^ + M
2. Dissociative electron attachment	AX + *e* ^−^ ⟶ X^−^ + A
3. Ion pair formation	AX + *e* ^−^ ⟶ A^+^ + X^−^ + *e* ^−^

Electron attachment to neutral molecules is a resonance process since no electron is produced to carry away the excess energy. Thermal or near‐thermal electrons usually react by ternary electron attachment, while electrons with higher energies (up to 15 eV) attach dissociatively. Threshold energies for ion pair formation are above 15 eV, so this mechanism may play a role only in highly energetic discharge plasmas.

Primary anions can undergo ion‐molecule reactions of several types (Harrison 1992):

**Table:** 

1. Proton transfer	X^−^ + YH ⟶ Y^−^ + XH
2. Displacement and elimination reactions	X^−^ + RY ⟶ Y^−^ + RX
3. Electron transfer	X^−^ + A ⟶ A^−^ + X
4. Ternary association reaction	X^−^ + A + M ⟶ XA^−^ + M
5. Associative detachment	X^−^ + A ⟶ XA + *e* ^−^

All these reactions proceed effectively when they are exothermic, forming more stable anions. The exception is associative detachment, which effectively removes the reagent anions.

##### 3.2.1.1 Formation of O^−^

In a typical DBDI experimental setup, gases such as nitrogen and air are used, often with the presence of traces of water vapor. Thus, the O^−^ radical anion can only be formed by the interaction of electrons with O_2_ or H_2_O molecules. When oxygen is considered, there are two possible mechanisms of O^−^ formation:

1. dissociative electron attachment to O_2_
(34) $$O_{2}+e^{-}\to O^{-∙}+O$$
2. ion pair formation:

(35) $$O_{2}+e^{-}\to O^{+∙}+O^{-∙}+e^{-}$$

The formation of negative ions by electron impact of O_2_ was reported already in the late 1950s (Frost and McDowell 1958).

The dissociative electron attachment to O_2_ showed a broad peak at 6.5 eV (Schulz 1962; Rapp and Briglia 1965) with a corresponding cross‐section of 1.3 × 10^−18^ cm^2^ (Schulz 1962). The ion pair formation mechanism in O_2_ was reported in 1965 (Rapp and Briglia 1965), where the total cross‐section for negative ion formation by electron impact was measured in the range of 4–55 eV. The dissociation dynamics of this process were studied by the velocity map imaging technique (Nandi, Prabhudesai, and Krishnakumar 2006; Nag and Nandi 2018) and the threshold of 15.3 eV for the O^+⋅^ + O^−⋅^ formation was determined (Nag and Nandi 2018). Thus, in the discharge plasma, the O^−^ ions are formed by the interaction of O_2_ with electrons of two energy domains; 4‐9 eV and > 15.3 eV. In the low energy domain, the O^−⋅^ ions are most likely formed by dissociative attachment of secondary electrons stemming from the electron ionization of neutral molecules, while the ion pair formation mechanism is dominant for more energetic primary plasma electrons. The cross‐section for the latter process with 30‐55 eV electrons is approximately one‐third of the value for the dissociative electron attachment at 6.5 eV.

In the discharge in an oxygen‐containing gas, the long‐lived singlet excited states can be formed. The dissociative electron attachment to the ^1^∆_g_ state of O_2_ was shown to process via two different states of O_2_^−⋅^, ^2^Π_u_, and ^2^Σ_g_^+^ (Jaffke et al. 1992). The cross sections for the O^−⋅^ formation via these two states were found peaking at 5.5 and 7.5 eV and larger than that for the ground triplet state of O_2_. Thus, the presence of singlet O_2_ broadens the above‐mentioned low‐energy domain and enhances the probability of O^−^ formation by dissociative electron attachment.

##### 3.2.1.2 Formation of O_2_^−^

It is well known that electron attachment to molecular oxygen at low electron energies (< 1 eV) is a three‐body process (reaction 36). This was first proposed as early as 1935 (Bloch and Bradbury 1935) and proved later on by electron swarm (Chanin, Phelps, and Biondi 1962) and electron beam (Spence & Schulz 1972) experiments.

(36) $$O_{2}+e^{-}+M\to O_{2}^{-∙}+M$$

The thermal rate coefficient for the reaction (36) in pure oxygen (M = O_2_) at 300 K was determined to be 2.0 to 2.8 × 10^−30 ^cm^−6^ s^−1^ (Chanin, Phelps, and Biondi 1962; Pack and Phelps 1966). The efficiency of nitrogen as a third body (M = N_2_) was found 50 times lower than for O_2_ (Chanin, Phelps, and Biondi 1962).

##### 3.2.1.3 Discharges in Air

The formation of negative ions in corona discharge in air was the subject of several studies (Gravendeel and Hoog 1987; Skalny et al. 2004; Sabo et al. 2010; Hiraoka et al. 2021). At low pressures, the ions NO_2_^−^, NO_3_^−^, and O_3_^−^ were found to dominate in dry air (Shahin 1969). However, their abundances decrease with increasing pressure due to the reaction with CO_2_ so that at pressures above 50 Torr (6.7 kPa), the CO_3_^−⋅^ ions become dominant and at atmospheric pressure, CO_3_^−⋅^ ions amount to > 90% of the total negative ion current (Shahin 1969). Later, different results were obtained when CO_3_^−⋅^ was found dominant already at pressures 10–30 Torr^17^ and 5 kPa (37.5 Torr) (Gravendeel and Hoog 1987). An appreciable amount of other ions (O^−⋅^, O_3_^−⋅^, and CO_4_^−⋅^) was detected together with CO_3_^−⋅^, and differences with previous work were ascribed to different amounts of trace water vapor in the “dry air” used for experiments (Gravendeel and Hoog 1987). Depending on the method, at least a part of the CO_2_ may be removed together with water vapor, which leads to a reduction of CO_3_^−⋅^ yield (Shahin 1969). When synthetic air containing ~5 ppm of water vapor and < 0.1 ppm of CO_2_, the ions O_3_^−⋅^, OH^−^, NO_3_^−^, and CO_3_^−⋅^ with relative intensities of 41%, 39%, 14%, and 6%, respectively, were observed at atmospheric pressure (Skalny et al. 2004).

The ozone anions O_3_^−⋅^ can be produced by the following reactions (McDaniel et al. 1970):

(37) $$O^{-∙}+O_{3}\to O_{3}^{-∙}+O,\;k=5.3\times 10^{-10}{\text{cm}}^{3}s^{-1}$$

(38) $$O_{2}^{-∙}+O_{3}\to O_{3}^{-∙}+O_{2},\;k=3\times 10^{-10}{\text{cm}}^{3}s^{-1}$$

(39) $$O^{-∙}+O_{2}+M\to O_{3}^{-∙}+M,\;k\left(M=O_{2}\right)=8.6\times 10^{-31}{\text{cm}}^{6}s^{-1}$$

However, when the concentration of ozone in the discharge is negligible, the three‐body reaction (6) is the only potential source of O_3_^−⋅^ ions. The O^−⋅^ reactants stem from reactions (34) or (35).

In the presence of carbon dioxide, the CO_3_^−⋅^ ions are formed from O^−⋅^ and O_3_^−⋅^ by reactions (40) (Fehsenfeld and Ferguson 1974) and (41) (McDaniel et al. 1970).

(40) $$O^{-∙}+{\text{CO}}_{2}+M\to {\text{CO}}_{3}^{-∙}+M,k\left(M=O_{2}\right)=3.1\times 10^{-28}{\text{cm}}^{6}s^{-1}$$

(41) $$O_{3}^{-∙}+{\text{CO}}_{2}\to {\text{CO}}_{3}^{-∙}+O_{2},\;k=4.0\times 10^{-10}{\text{cm}}^{3}s^{-1}$$

The CO_4_^−⋅^ ions can be formed from O_2_^−⋅^ (see reaction 36) (Fehsenfeld and Ferguson 1974).

(42) $$O_{2}^{-∙}+{\text{CO}}_{2}+M\to {\text{CO}}_{4}^{-∙}+M,\;k(M=O_{2})=4.7\times 10^{-29}{\text{cm}}^{6}s^{-1}$$

The O_2_^−⋅^ ions can also react with oxygen molecules to form rather unstable ions O_4_^−⋅^ that can easily dissociate back to O_2_^−⋅^ or convert to CO_4_^−⋅^ (McDaniel et al. 1970; Payzant and Kebarle 1972).

(43) $$O_{2}^{-∙}+O_{2}+M\to O_{4}^{-∙}+M,\;k(M=O_{2})=5.1\times 10^{-31}{\text{cm}}^{6}s^{-1}$$

(44) $$O_{4}^{-∙}+{\text{CO}}_{2}\to {\text{CO}}_{4}^{-∙}+O_{2},\;k=4.3\times 10^{-10}{\text{cm}}^{3}s^{-1}$$

The population of ions CO_4_^−⋅^ is, however, reduced when ozone or oxygen atoms are present in the discharge gas (McDaniel et al. 1970; Fehsenfeld and Ferguson 1974).

(45) $${\text{CO}}_{4}^{-∙}+O_{3}\to O_{3}^{-∙}+{\text{CO}}_{2}+O_{2},\;k=1.3\times 10^{-10}{\text{cm}}^{3}s^{-1}$$

(46) $${\text{CO}}_{4}^{-∙}+O\to {\text{CO}}_{3}^{-∙}+O_{2},\;k=2.0\times 10^{-10}{\text{cm}}^{3}s^{-1}$$

(47) $$\;\to O_{3}^{-∙}+{\text{CO}}_{2},\;k<1.5\times 10^{-10}{\text{cm}}^{3}s^{-1}$$

The neutral reactants in reactions (45)–(47), O and O_2_, are formed in the DC corona discharge in ambient air by electron‐induced dissociation (48) and subsequent ternary association (49) (Atkinson et al. 1997).

(48) $$O_{2}+e^{-}\to O+O+e^{-}$$

(49) $$O+O_{2}+M\to O_{3}+M$$

No reliable data are available for the total cross‐section for dissociation of O_2_; however, there are at least four excited states for which only the repulsive part of the potential energy curve lies in the Franck–Condon region of the ground state and thus, an excitation to any of these states mostly results in dissociation (Itikawa et al. 1989).

Oxygen atoms are also formed by the above‐mentioned dissociative electron attachment (34) and by dissociative electron ionization of O_2_, provided the electrons with sufficient energy are present in the plasma.

(50) $$O_{2}+e^{-}\to O^{+}+O+2e^{-}$$

The cross‐section for the reaction (50) has an onset at 18.734 eV (Blyth, Powis, and Danby 1981) and reaches a value of 1 × 10^−16 ^cm^2^ at ~150 eV (Itikawa et al. 1989).

Similarly, nitrogen oxides are formed from molecular nitrogen. The first step in this process is the formation of nitrogen atoms, which can be formed by the following electron‐induced reactions.

(51) $$N_{2}+e^{-}\to N^{∙}+N^{∙}+e^{-}$$

(52) $$N_{2}+e^{-}\to N^{+}+N^{∙}+2e^{-}$$

The cross sections for reactions (51) and (52) were measured (Rapp, Englander‐Golden, and Briglia 1965; Winters 1966) and thoroughly reviewed (Itikawa et al. 1986).

The total dissociation cross‐section, that is, for both reactions, was found to have a maximum value of 2 × 10^−16 ^cm^2^ at an electron energy of 90 eV (Winters 1966), and the contribution of reaction (52) is approximately one‐third of the total (Rapp, Englander‐Golden, and Briglia 1965; Itikawa et al. 1986).

Nitrogen atoms then react with O_2_ to form nitric oxide, ^⋅^NO (Burkholder et al. 2019).

(53) N∙+O2→NO∙+O,k=8.5×10−17cm3s−1

The rather small rate coefficient for the reaction (53) involving ground state N(^4^S) dramatically increases to *k* = 5.2 × 10^−12^ cm^3^ s^−1^ and *k* = 2.5 × 10^−12^ cm^3^ s^−1^ for N(^2^D) and N(^2^P) excited states, respectively (Herron 1999).

From ^⋅^NO, the nitrogen dioxide, ^⋅^NO_2_, and nitrogen trioxide, ^⋅^NO_3_, can be formed by several neutral‐neutral reactions as reviewed in a recent surface DBD study (Kim et al. 2023). However, due to their low cross sections and low concentrations of the reactants involved, the ionic pathways leading to NO_2_^−^ and NO_3_^−^ seem to be more likely than electron attachment or electron transfer to neutral ^⋅^NO_2_ and ^⋅^NO_3_. The only exception may be the formation of ^⋅^NO_2_ by the ternary association reaction (54) (Atkinson et al. 1997).

(54) O+NO∙+M→ON∙2+M,k(M=N2)=1.0×10−31cm3s−1

Associative electron detachment reactions can also contribute to formation of neutral NO_x_ molecules (McFarland et al. 1973; Fehsenfeld and Ferguson 1974):

(55) O−∙+N∙→NO∙+e−,k=2.2×10−10cm3s−1

(56) O−∙+NO∙→O2∙N+e−,k=2.5×10−10cm3s−1

(57) O2−∙+N∙→O2∙N+e−,k=4.0×10−10cm3s−1

(58) →NO∙+O+e−,k=4.0×10−10cm3s−1

The neutral ^⋅^NO_x_ molecules can be transformed into negative ions by several ion‐molecule reactions, such as (Rutherford and Turner 1967; Fehsenfeld and Ferguson 1974):

(59) O−∙+N∙O2→NO2−+O,k=1.2×10−9cm3s−1

(60) O2−∙+N∙O2→NO2−+O2,k=8.0×10−10cm3s−1

(61) O3−∙+NO∙→NO3−+O,k=1.0×10−11cm3s−1

(62) O3−∙+N∙O2→NO3−+O2,k=2.8×10−10cm3s−1

(63) $$\to {\text{NO}}_{2}^{-}+O_{3},\;k=7.0\times 10^{-10}{\text{cm}}^{3}s^{-1}$$

(64) CO3−∙+NO∙→NO2−+CO2,k=0.9×10−11cm3s−1

(65) CO3−∙+N∙O2→NO3−+CO2,k=2.0×10−10cm3s−1

##### 3.2.1.4 Influence of Water Vapor

A mass spectrometric study of negative ions in corona discharge in ambient air showed that at specific conditions, hydrates of O_2_^−^, O_3_^−^, NO_2_^−^, NO_3_^−^, and CO_3_^−^ were formed and observed (Skalny et al. 2008). A sequence of ternary association reactions was suggested to be responsible for these observations.

(66) $$X^{-}{\left(H_{2}O\right)}_{n}+H_{2}O+M\to X^{-}{\left(H_{2}O\right)}_{n+1}+M\;(n=0,\;1,\;2,\;\text{…})$$

The rate coefficients have been measured only for unhydrated X^− ^= CO_3_^−⋅^, O_3_^−⋅^, NO_2_^−^, M = O_2_ (*n* = 0) and are in the order of 10^−28 ^cm^6 ^s^−1^; reactions for n > 0 are expected to be similarly fast (Skalny et al. 2008).

For X^− ^= NO_2_^−^ and NO_3_^−^, alternative processes were assessed as more likely:

(67) O2−∙(H2O)n+NO∙2→NO2−(H2O)n−1+O2+H2O

(68) CO3−∙(H2O)n+N∙O2→NO3−(H2O)n+CO2

In the same study, hydrated OH^−^ ions were also observed. Their origin was ascribed to the following sequence of reactions.

(69) $$O^{-∙}+H_{2}O+O_{2}\to O^{-∙}\left(H_{2}O\right)+O_{2}$$

(70) O−∙(H2O)+H2O→OH−(H2O)+OH∙

(71) $${\text{OH}}^{-}\left(H_{2}O\right)+H_{2}O+M\to {\text{OH}}^{-}{\left(H_{2}O\right)}_{2}+M$$

The negative ion chemistry in discharges in air is apparently more complex than positive ion chemistry, and the populations of the above‐mentioned ions strongly depend on the quality of discharge gas (i.e., the presence of trace impurities, such as CO_2_ and water vapor) and on the technical parameters and design of the discharge cell.

#### 3.2.2 Product Ions in Negative Mode

Negative‐ion mass spectra typically contain the radical anion M^−⋅^, deprotonated molecule [M − H]^–^ anions, or adducts [M + NO_2_]^−^. Oxidized species such as [M – X + O]^−^ were also observed (Cody, Laramée, and Durst 2005; Gyr et al. 2018). The formation of stable negative ions requires the presence of electronegative elements in the molecule, including O, like COOH, in acidic groups or halogens. Below is a summary of the processes involved in their formation.

##### 3.2.2.1 Electron Attachment

The free electrons formed in DBD can react by electron attachment. In practice, free electrons can survive for more than a microsecond only when the O_2_ concentration is less than 1% and may thus play a role in clean N_2_ or He carrier gases. Depending on the nature of the analyte, ternary electron attachment can form a radical molecular anion:

(72) $$M+e^{-}+N_{2}\to M^{-∙}+N_{2}$$

Or when exothermic, dissociative electron attachment can form fragment ions: (de Hoffmann and Stroobant 2007)

(73) $$M+e^{-}\to {\left(M-B\right)}^{-}+B^{∙}$$

At higher O_2_ concentrations, free electrons will be converted to superoxide anions O_2_^–⋅^ which will act as reagent ions for chemical ionization of analytes.

##### 3.2.2.2 Electron Transfer

Electron transfer reaction from O_2_^−⋅^ to analyte molecules M will take place when the electron affinity of M is higher than the electron affinity of O_2_ (0.45 eV), forming molecular radical anion M^•−^ or its characteristic fragments:

(74) $$O_{2}^{-∙}+M\to M^{-∙}+O_{2}$$

(75) $$\;\to {\left[M-X\right]}^{-}+X^{∙}+O_{2}$$

Where in the case of halogenated analytes X = F, Cl, or Br (Gyr et al. 2018). In the case of nitro compounds, for example, explosives X = ^⋅^NO, ^⋅^OH, or ^⋅^NO_2_, (Usmanov et al. 2013; Plasmion 2020).

##### 3.2.2.3 Proton Transfer

O_2_^−⋅^ has strong gas‐phase basicity and may thus extract the proton from molecules with gas‐phase acidity lower than the neutral product of this reaction HO_2_^⋅^, 1451 kJ/mol: (de Hoffmann and Stroobant 2007)

(76) $$O_{2}^{-∙}+M\to {\left[M-H\right]}^{-}+{\text{HO}}_{2}^{∙}$$

This is a typical process for many oxygen‐containing organic molecules, including carboxylic acids or phenolic compounds (Basham et al. 2022).

##### 3.2.2.4 Substitution Reactions

The reactions of O_2_^•−^ can also proceed as a substitution of a hydrogen, halogen, or nitryl group by oxygen to produce oxidation products: (Dousty and O'Brien 2015; Gyr et al. 2018)

(77) $$O_{2}^{-∙}+M\to {\left[M-X+O\right]}^{-}+{\text{OX}}^{∙},\text{where}X=H,F,\text{Cl},\text{Br},{\text{NO}}_{2}.$$

Such product ions were observed for perfluorinated compounds (Gyr et al. 2018).

##### 3.2.2.5 Ternary Association

The NO_2_^–^ and NO_3_^–^ reagent anions can associate with analyte molecules forming adduct ions [M + NO_2_]^−^ and [M + NO_3_]^−^:

(78) $${\text{NO}}_{2}^{-}+M+N\to {\text{MNO}}_{2}^{-}+N$$

(79) $${\text{NO}}_{3}^{-}+M+N\to {\text{MNO}}_{3}^{-}+N$$

This was observed, for example, in case studies of explosives (Harper et al. 2008; Gilbert‐López et al. 2013).

## 4 Sample Introduction Methods

The growing popularity of DBDI is also due to the relative ease of quick analyses of a wide variety of sample forms or matrices. For the sake of a concise discussion, the individual studies summarized in this section are categorized according to the method by which the analyte molecules are vaporized and introduced into the plasma of the ion source. Some studies were carried out using commercial DBDI sources, and some were also carried out using bespoke DBDI sources constructed in various laboratories; this will be indicated for each study.

**Figure 5:**
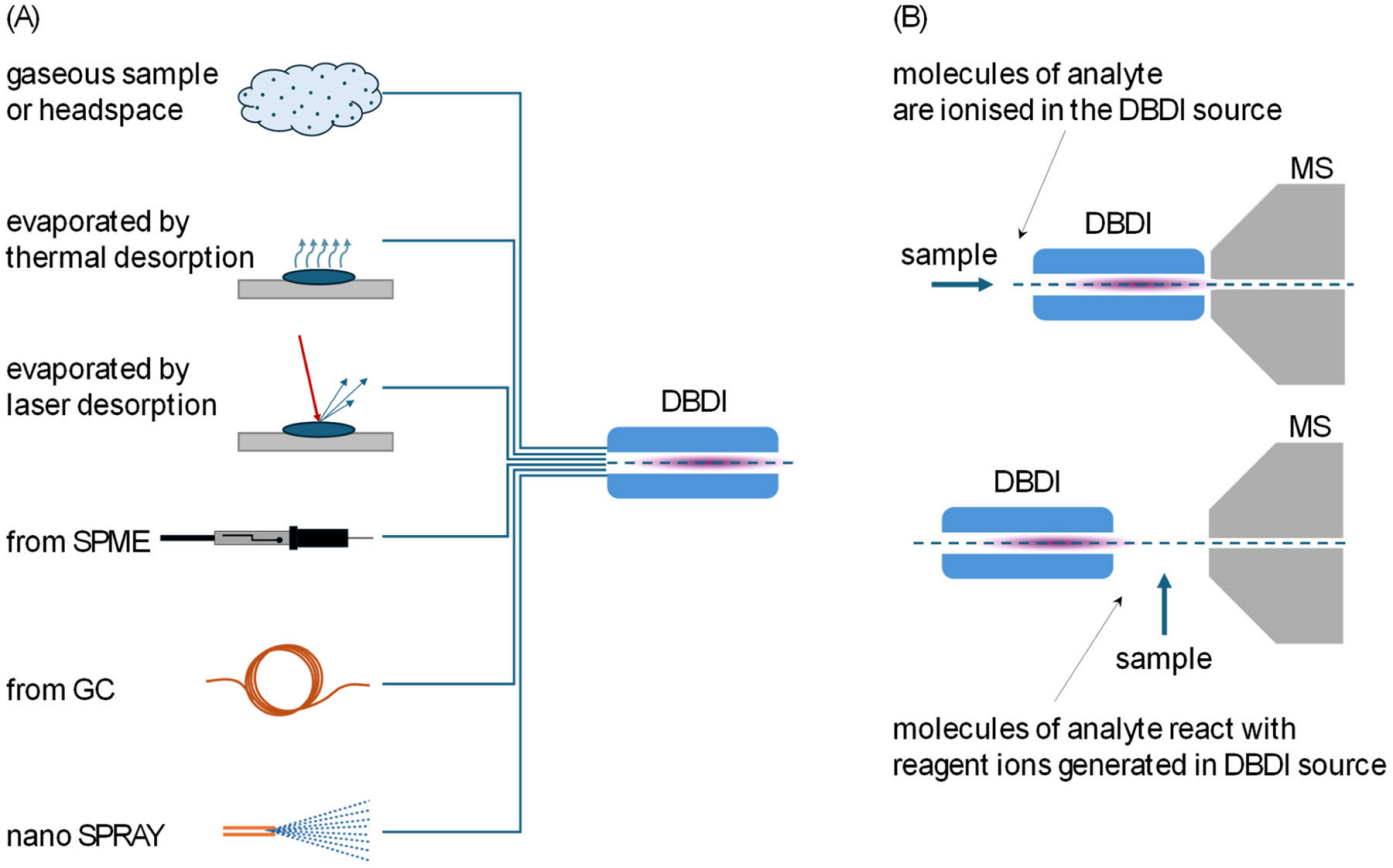
The different ways of sample introduction into DBDI‐MS. (A) Possible origins of analyte molecules introduced to DBDI. (B) Arrangements where the sample goes through the DBDI or where it is introduced to a plasma formed from clean discharge gas. [Color figure can be viewed at wileyonlinelibrary.com]

The multiple ways of sample introduction are indicated in Figure 5. The simplest method involves just the introduction of a gaseous sample to the ion source. Analytes present on the surface of solid, liquid, or biological samples can be desorbed by heating (evaporation by thermal desorption) or by laser‐induced desorption. Another method that was used for liquid solutions involves vaporization by nano spray (either with or without LC). Solid‐phase microextraction using a sorbent‐coated fiber was also successfully combined with DBDI. Finally, DBDI can be used to ionize eluent from GC columns.

Once the sample is vaporized and available as atmospheric pressure gas, can be introduced directly into the DBD, where it is ionized (Massaro et al. 2024), this is the case for the current commercial ion sources. Alternatively, the sample can be introduced to a reaction zone after DBD without going through the active discharge (see Figure 5B), and analyte molecules are ionized by cold plasma before transfer to the mass analyzer (Saha et al. 2013).

Depending on the requirements of a specific study, samples can be introduced in an open arrangement, where the ion source draws in the ambient laboratory air and samples or their headspace are simply placed in front of the ion source inlet (Raeber and Steuer 2023). This obviously means that many impurities, including ammonia and VOCs, will be present in the discharge. To avoid this, some studies used closed arrangements with clean carrier gases (air or nitrogen) flowing over the sample (Hiraoka et al. 2011).

In this review, we will only cover the studies where the gaseous sample interacts with the plasma and will not refer to articles describing DBD plasma interacting with the sample surface (Na et al. 2007) in the so‐called “ambient MS” approaches. These studies are covered in reviews by Joachim Franzke (Brandt et al. 2017) and Xinrong Zhang (Guo et al. 2015b) and their colleagues.

### 4.1 Gaseous Samples or Headspace

Gaseous samples could be directly introduced into the DBDI source together with carrier gas. An interesting recent example of the use of the SICRIT ion source for breath analysis was presented at the 2023 ERS International Congress (Abuhelal et al. 2023). Asthmatic patients and healthy volunteers exhaled into the entrance of the SICRIT ion source coupled to HRMS. Several volatile metabolites were identified from ion peaks in the *m*/*z* range of 100–250, including pyruvate, urea, and lactate. Some metabolites (aldehydes) were linked to asthma and a change between rest and exercise was observed.

A good example of headspace analyses using the commercial SICRIT source connected to a quadrupole‐Orbitrap mass spectrometer was a study of VOCs released by 13 filamentous fungi belonging to *Aspergillus* sp., *Ceratocystis* sp., and *Neurospora* sp. (Heffernan et al. 2023). These fungi were cultured in liquid media, and it was found that ambient sampling where the open vials were placed directly in front of the DBDI source was superior in comparison with sampling from a closed vial via septum or using a syringe. All eight standards used for targeted analyses, including esters, alcohols, and aldehydes, led to mass spectra dominated by fragments. VOCs from fungal samples in three different types of complex growth media show clear differences in VOC profiles across the different media, enabling the determination of the best culturing conditions for each compound‐strain combination. However, the authors concluded that this method is not suitable for untargeted analyses of fungal emissions.

Another nice example of the application of DBDI‐MS to food science is a recent study of the origins of honey (Massaro et al. 2024). SICRIT ion source combined with an Orbitrap MS was used for rapid analyses of the headspace of 112 honey samples with seven different botanical origins (acacia, dandelion, chestnut, rhododendron, citrus, sunflower, and linden). The sample headspace was simply introduced to the inlet of the SICRIT source by holding an open vial nearby. The conclusion was that authentication of monofloral honey is possible using this method. Another paper demonstrates the potential of SICRIT source with QTOF mass spectrometry for rapidly distinguishing dairy products with protected geographical indications based on differences in spectral features, as analyzed by PCA, between Hongyuan yak milk and the other three origins (Zhang et al. 2024). The putative assignment of the peaks was done on the assumption that they are protonated molecules.

### 4.2 Thermal Desorption

This method is used for samples that are not in the gaseous matrix or involve semivolatile analytes. One of the first papers on this subject presented a combination of a bespoke DBDI source with closed heated sample containers (Hiraoka et al. 2011). A sample holder with a liquid sample was put inside the sample container (see Figure 2A). After the sample vial was placed, the temperature of the sample holder was raised from room temperature to 150°C. Methamphetamine could then be detected with a LOD of a few picograms.

The flow through the DBDI source allows for easy connection to a thermal desorption sample introduction device. For example, a recent study demonstrated the identification of persons based on the chemical profile of their fingerprints using a bespoke closed thermal desorption device (Conway et al. 2023). In this device, the sample, introduced via a glass slide, was heated and flushed by dry N_2_. The evaporated compounds were then transferred into the SICRIT‐DBDI‐MS for identification and semi‐quantification. The combination of thermal desorption and DBDI‐MS minimized sample preparation, leading to an ultrasensitive and rapid analysis of illicit drug traces and the identification of individuals based on fingerprints.

Another study compared concave and flat thermal desorption methods in open and semi‐covered configurations for rapid detection of fentanyl analogs in dried blood and plasma spot samples (Li et al. 2022). In this study, the DBDI‐100 source operated with pure helium as the discharge and carrier gas. Analytes evaporated in the thermal desorption device were introduced into a separate reaction chamber between the DBDI source and mass analyzer to be ionized by cold plasma. The semi‐covered flat‐TD surface method of sample introduction was shown to be optimal for screening fentanyl derivatives in 20 s for 10 μL of plasma or blood.

Recent studies of particulates in exhaust gas (Thaler et al. 2021; Gelner et al. 2022) were performed using a “high‐efficient light source for optical surface desorption,” principally an infrared oven used for the evaporation of volatile particle components.

### 4.3 Laser Desorption

The combination of the DBDI source and laser desorption allows for the visualization of the spatial distribution of numerous compounds from a complex sample surface in a single experiment. Laser desorption in combination with a custom‐made FμTP ionization source was used for the first time and applied to detection of hydrophobic compounds including cholesterol and other lipids (Knodel et al. 2020a; Knodel et al. 2020b).

**Figure 6:**
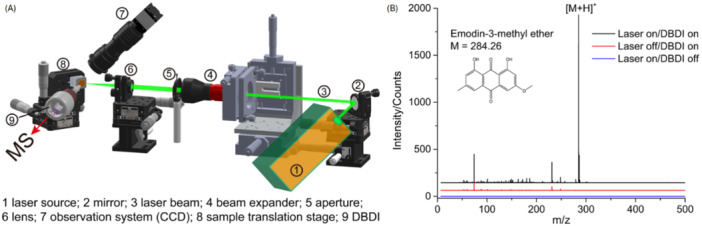
(A) Schematic diagram of the laser desorption – DBDI‐MS imaging apparatus. (B) Mass spectra of background and desorbed emodin‐3‐methyl ether. Adapted from Xiao et al. (2024) licensed under CC‐BY 4.0. Copyright 2024 by original authors. [Color figure can be viewed at wileyonlinelibrary.com]

For instance, it was recently used to image the in‐situ metabolic profiling of two types of plants: a wild and a farmed Chinese rhubarb (Xiao et al. 2024). In this study, the sample was vaporized by laser desorption, leading to the formation of a gaseous plume above the sample. Subsequently, the gaseous sample molecules were drawn into the custom‐made DBDI source and, after ionization, analyzed by a Q‐TOF MS system, see Figure 6A. In this study, the efficiency of the DBDI source was also tested with and without laser desorption using standard emodin‐3‐methyl ether, which was compressed into a pellet using a tablet press and coated onto the sample plate. The blue trace in Figure 6B reveals that no distinct mass spectral signals were observed when the ionization source was deactivated, even though the desorption laser was triggered. This indicates that a single desorption laser, without post‐ionization, fails to generate a meaningful signal contribution in this experiment. The red trace obtained without laser by the active DBDI source represents the lab air background, such as plasticisers and volatile reagents. The black trace illustrates that the characteristic peak of emodin‐3‐methyl ether (*m*/*z* 285) appeared only when both the desorption laser and DBDI source were activated.

UV‐laser ablation combined with SICRIT SC‐30 DBDI ionization set and an Exactive Orbitrap mass spectrometer was used for the direct, molecule‐specific, and spatially resolved analysis of various solid samples, such as coffee beans and painkiller tablets (Funke et al. 2021). As in the previous study, the vaporized analytes were subsequently ionized with DBDI. This combination of fast washout UV‐laser ablation and DBDI allowed for highly efficient soft ionization, high spatial resolution of 10 μm for molecular imaging, and short analysis times.

The potential of DBDI for MS imaging was also demonstrated in several studies from Zenobi's group: standard samples with a striped pattern, sections of fingernails treated with the drug methyl green zinc chloride salt (Lu et al. 2021a) and on endogenous species in traditional Chinese herbal medicine and of a drug molecule in zebra fish tissue, with a lateral resolution of ≈20 μm (Lu et al. 2021b).

### 4.4 Solid Phase Microextraction

Solid phase microextraction (SPME), based on the thermal desorption of analytes extracted from the fibers, is a well‐established technique for sampling preconcentration, storage and transportation for the subsequent analysis. It is widely used in combination with different MS instruments. A direct coupling of SPME with DBDI‐MS was first reported in 2016 by Renato Zenobi's group (Mirabelli, Wolf, and Zenobi 2016). The method was validated on several drugs, including diazepam, cocaine, atrazine, ametryn, parathion, and triethyl thiophosphate using the 65 μm PDMS/DVB and the 100 μm PDMS fibers. Limits of detection as low as 0.3 pg/mL and a linear dynamic range of more than three orders of magnitude were achieved.

The combination of SPME with DBDI‐HRMS was shown to be very effective for the analysis of pesticides in grape juice, a rather complex matrix due to the high content of sugars and pigments (Mirabelli et al. 2018). The analytes were thermally desorbed from the SPME fibers in a lab‐built stainless‐steel desorption chamber and introduced in neutral form into the ionization source together with humidified nitrogen. For this study, the desorption chamber was modified to reduce the adsorption of analytes desorbed from the fiber onto the metal walls of the desorption chamber. Therefore, glass capillaries were placed in the inner diameter of the chamber, the walls of which were passivated by salinization.

A similar direct and fast method for screening illicit drugs in beverages and biological fluids was developed by using extraction of the targeted analytes with thin film microextraction (Mirabelli et al. 2019). The targeted analytes were then thermally desorbed and introduced into the DBDI source without the need for any cryofocusing apparatus. The proposed method was shown to have enhanced performances when compared to a conventional TDU‐GC‐EI‐MS approach for eight drugs extracted from biological fluids (urine and blood plasma) and beverages (vodka, cola, and wine).

In another study (Dumlao et al. 2016), a custom‐fabricated SPME fiber was directly integrated with an ACaPI source to detect organophosphate chemical warfare agent simulants and their hydrolysis products in seawater and urine with detection limits below 100 ppbv for 2 min analyses per sample.

A direct, fully automated coupling approach of SPME autosampler to a commercially available SICRIT SC‐10 and HRMS was presented for polar but importantly also for non‐polar analytes like a broad range of contaminants in environmental water samples in (Huba, Mirabelli, and Zenobi 2018).

### 4.5 Gas Chromatography

An LTP ionization interface between a gas chromatograph and an atmospheric pressure inlet mass spectrometer was first described by Nørgaard et al. (2013). Twenty different common indoor VOCs with eight functional groups (alkanes, alkenes, alcohols, aromatic compounds, aldehydes, PAHs, phenols, and terpene alcohols) were analyzed. Analyte molecules separated in GC were ionized by plasma generated in pure helium.

In the study carried out by Zenobi's group (Mirabelli, Wolf, and Zenobi 2017), a DBDI source was employed to ionize GC‐resolved pesticides and illicit drugs. Because in conventional GC experiments, the column flow ranges between 1 and 3 mL/min, a curtain gas of humidified N_2_ (90% R.H. at 25°C) was used to maintain this required total gas flow entering the DBDI source. The simplicity of the proposed approach without the requirement of dedicated vacuum interfaces and the absence of strict geometric requirements to interface the GC column to the DBDI source were considered of high practical importance.

Another study (Vogel et al. 2019) was focused on the interaction between the DBDI plasma, certain analytes introduced via gas chromatography, and the surrounding atmosphere. To define a controlled atmosphere, the plasma was connected to the mass spectrometer using a closed reactant capillary supplied by a reactant gas. Different reactant gases (Ar, He, O_2_, and N_2_) and reactant gas mixtures were tested to optimize the DBDI performance and to improve the ionization efficiency for perfluorinated compounds.

Gas chromatography using the SICRIT source coupled to an HRMS was described in (Weber et al. 2021). The interface with the MS was done via a commercially available GC/SPME module with a heated transfer line (Plasmion GmbH, Augsburg, Germany) in a setup similar to that described by (Mirabelli, Wolf, and Zenobi 2017). The method was validated on n‐alkanes from decane to triacontane and then applied to a real diesel fuel sample. Calibration curves for n‐alkanes show high linearity, reproducibility, and LODs in the low parts per billion (ppb) range.

In a recent study, (Weber et al. 2023) investigated ionization in SICRIT using eight different makeup gas compositions (dry nitrogen, room air, and nitrogen enriched with water, HCl, methanol, hexane, NH_3_, and fluorobenzene). Gas chromatography SICRIT high‐resolution mass spectrometry (GC‐SICRIT‐HRMS) data were obtained for fifteen compound classes: alkanes, polyaromatic hydrocarbons, terpenes, oxygen‐containing terpenes, alkylphenols, chlorophenols, nitrophenols, trialkyl amines, triazines, phthalates with or without ether groups, aldehydes, ketones, fatty acid methyl esters, and polyoxyethylene ethers. The study serves as a guideline for the choice of atmosphere for specific compound classes and the interpretation of spectra generated with specific makeup gases and dopants.

### 4.6 Nebulization, Nanoelectrospray

In addition to previously described methods, liquid samples can be analyzed by DBDI‐MS using forced evaporation or spraying. The first coupling of an atmospheric pressure microplasma ionization source based on DBD with liquid chromatography was published in 2009 (Hayen et al. 2009). For these purposes, a modified Ion Max (Thermo) API source was used. The front window of the Ion Max source was replaced by the DBDI probe head with a helium plasma cone outside the electrode region. Nitrogen was used to nebulize the liquid eluent with several model analytes covering a wide range of polarities, including polar compounds like threonine, tryptophan, and glutamic acid and nonpolar compounds like PAHs.

Another configuration of the commercial Ion Max source was used later (Gilbert‐López et al. 2012), where a bespoke DBDI probe was fitted in place of APCI in an orthogonal orientation relative to the transfer capillary entrance of the mass analyzer. The combination of this ionization source with a fast polarity switching high‐resolution mass spectrometer enabled the simultaneous acquisition of both positive and negative ion modes of polar and nonpolar compounds in a single run with acquisition cycles matching the requirements of liquid chromatography. Different applications were outlined including the determination of pesticides, pharmaceuticals, and drugs of abuse in foodstuffs and in wastewater.

Later, a similar setup was used for lipid analyses, including triacylglycerides and sterols in archeological samples (Bouza et al. 2022). The lipids were extracted from samples of internal waterproof coverings of three archeological structures located in a Roman site (2nd–4th centuries AD) of Vilardida (Montferry and Vilarrodona, Tarragona, Spain). The coverings are made of mud and lime, and serve to waterproof the interior wall.

Nebulization of the liquid sample before introduction into the DBDI source was used for direct analysis of low‐polar acenaphthene in real water samples, including river water, initial rainwater, and mineral water collected from five different sites in Weihai, China (He et al. 2021). The sample microdroplets were generated using a bespoke flow injection sprayer and directly introduced into a heated glass tube with helium flow as a nebulization gas in line with the DBDI source. The use of nebulization and heating boosted ionization efficiency, and the high‐velocity gas from the sprayer directed to the MS inlet ensured high‐efficiency ion transmission without interference.

A hybrid ionization source that combines nanoelectrospray ionization and DBDI‐MS was proposed for single‐cell analysis (Liu et al. 2022). The combination of nanoESI with DBDI for the ionization of polar metabolites was employed to improve the ionization of polar metabolites in cells that are not easily ionized by ESI. Approximately 111 metabolites were detected in a single PANC‐1 cell when the DBDI was turned on, of which an additional 71 metabolites were not observed in ESI‐only mode.

## 5 Concluding Remarks

While the principle of dielectric barrier discharge has been known for more than a hundred years, it has only been used in mass spectrometry for soft ionization as DBDI for the last 17 years. Several configurations were developed in research laboratories around the world, often under varying names and abbreviations (like active capillary plasma ionization, ACaPI, in which the sample passes through the discharge introduced by Renato Zenobi; low‐temperature plasma, LTP; flexible microtube plasma, FμTP, and others). These techniques have been gaining popularity, especially in the last 8 years, since commercial ion sources (DBDI‐100 and SICRIT) based on these principles are available as add‐ons for standard mass analyzers. Considerable research efforts have been undertaken to optimize the analytical performance of these DBDI and to understand the ionization mechanisms and the trends in observed product ions on the mass spectra. It is thus now understood that the nature of the discharge gas is of primary importance; helium acts differently than nitrogen or air. It is also known that the presence of water vapor has a major effect on the ionization efficiency and the nature of the product ions. In the present review, we have summarized the ion chemistry involved in the formation of the reagent ions in typical DBDI configurations and outlined the principles of their reactivity with analyte molecules, focussing on gas‐phase chemistry when the sample is either gaseous naturally or is vaporized from liquids or solids.

Currently, DBDI‐MS methods are used in a range of applications, usually in a semiquantitative manner or using relative quantification based on standards. The matrix effects and competition of analytes for ionization are important because of the great sensitivity of this method. However, they are not fully accounted for routinely. The development of algorithms for the accurate quantification of gaseous analytes will surely extend the range of applications in the biological, environmental, and medical areas. One of the areas where future work can be directed is the development of robust absolute quantification calculation methods using the ratios of DBDI‐MS ion signals based on understanding the kinetics of ionization processes.

## Author Contributions

**Kseniya Dryahina:** conceptualization, formal analysis, funding acquisition, project administration, resources, writing–original draft, writing–review and editing. **Miroslav Polášek:** formal analysis, investigation, writing–original draft, writing–review and editing. **Juraj Jašík:** formal analysis, investigation, writing–original draft. **Kristýna Sovová:** formal analysis, investigation, writing–review and editing. **Patrik Španěl:** conceptualization, writing–original draft, writing–review and editing.
